# Harnessing inhomogeneous π-aggregates: a new path to optical modulation in methyl salicylate-based solvent-free liquids

**DOI:** 10.1039/d5sc06148b

**Published:** 2025-10-22

**Authors:** Kei Kobayashi, Ken-ichi Sakai, Tomoyuki Akutagawa, Takashi Nakanishi

**Affiliations:** a Graduate School of Science and Technology, Chitose Institute of Science and Technology (CIST) Chitose 066-8655 Japan k-sakai@photon.chitose.ac.jp; b Polymer Hybrid Materials Research Center, Institute of Multidisciplinary Research for Advanced Materials (IMRAM), Tohoku University Sendai 980-8577 Japan; c Research Center for Materials Nanoarchitectonics (MANA), National Institute for Materials Science (NIMS) 1-1 Namiki Tsukuba 305-0044 Japan

## Abstract

Liquids are characterized by macroscopic isotropy and homogeneity, yet they can exhibit the ability to self-organize at the molecular level. Driven by intermolecular forces such as coulombic and dispersion interactions, the constituent molecules spontaneously form locally heterogeneous structures. This behavior highlights the dynamic complexity and inherent adaptability of liquid systems. We found that methyl salicylate (MS), a colourless liquid, transforms into a vivid yellow liquid with distinct absorption and fluorescence spectral characteristics when an alkoxy chain with a carbon number of four to seven is attached to the 5-position of MS. This phenomenon suggests the presence of microscopic local structures in the liquids, where π-electrons are delocalized across multiple MS molecules. X-ray diffraction (XRD) measurements supported this interpretation by indicating aggregates in the 10–15 Å range, composed of about three to five MS molecules arranged in a face-to-face π-stacking configuration. When the alkylated MS liquids are heated from temperatures below their glass transition temperature, they exhibit the property of crystallizing from a supercooled liquid state, known as cold crystallization. Furthermore, free-volume analysis using positron annihilation lifetime spectroscopy (PALS) suggested that the yellow liquids have fewer free-volume regions compared to the unsubstituted MS colourless liquid. Consistent with these findings, time-resolved fluorescence spectroscopy of the neat yellow liquids revealed a relatively longer lifetime of 20 ns, which is likely attributable to fluorescence from π-aggregates, distinct from the 0.5 ns shorter lifetime component observed for single molecules in diluted solutions. All of these results support the presence of π-aggregates, which contribute to the colour and fluorescence of the liquids. Supramolecular aggregates in liquids have been studied both experimentally and theoretically; however, the alkoxylated MS liquids represent the first example in which they significantly affect the optical properties of the liquids.

## Abstract 2

Alkoxylated methyl salicylate liquids (C_4_–C_7_) exhibit vivid yellow coloration and strong fluorescence arising from inhomogeneously distributed π-aggregates in the liquids.

## Graphical abstract

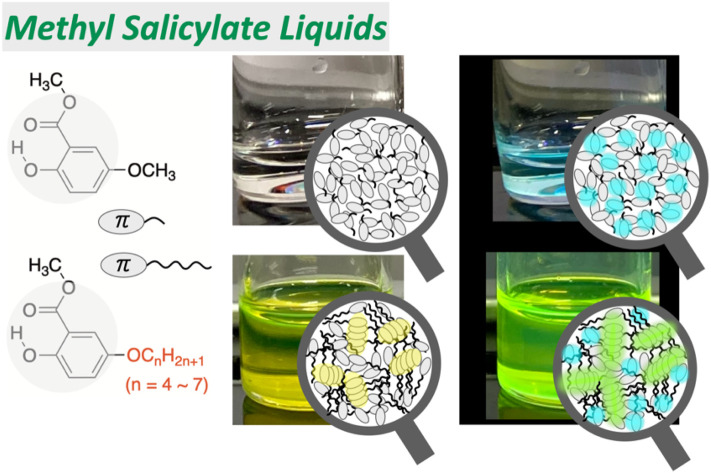

## Introduction

In condensed systems of π-electronic molecules, the arrangement of the molecules is closely related to the functionalities they offer; particularly, the carrier transport properties are significantly influenced by intermolecular interactions, such as vertical π–π overlaps and side-by-side sulfur–sulfur contacts.^1^ Peripheral modification of π-molecules with linear alkyl chains is an effective strategy for controlling molecular arrangement. This approach can enhance intermolecular interactions, contributing not only to the development of high-performance organic devices and their facile fabrication,^2,3^ but also to the design of liquid crystal molecules, especially by improving alignment properties and stabilizing the liquid crystal phase.^4^ Meanwhile, such modifications contribute to increasing solubility of π-molecules to solvents, thereby facilitating better processability using solution processing methods. Although bulkier modifications, such as the use of branched alkyl chains, can rather act as obstacles to intermolecular π–π interactions, they can also offer a new category of materials called molecular liquids, which are anticipated to have promising uses for soft electronics.^5–9^

On the other hand, it is rather challenging to derive optical properties from condensed systems of π-molecules. Regarding fluorescence properties, intermolecular interactions tend to cause fluorescence quenching, and in fact, examples of organic crystals showing high fluorescence are limited.^10^ Aggregate-induced emission enhancement (AIEE) is a phenomenon that addresses this quenching problem,^11^ but the emission does not come from molecular aggregates; rather, it comes from individual molecules whose intramolecular motions are restricted by aggregation. Thus, fluorescence from excited states involving multiple π-molecules is uncommon. An exception is fluorescence from J-aggregates composed of head-to-tail molecular arrangements, which results from intermolecular exciton coupling among multiple π-molecules.^12^ Such J-aggregates exhibit unique absorption and fluorescence spectra, showing bathochromic shifts, sharpened linewidths, and much smaller Stokes shifts compared to those of individual chromophores. Introduction of linear alkyl/alkoxy chains to chromophores can be effective to construct J-aggregates.^13^ To the best of our knowledge, no other examples of aggregate-based fluorescence like that seen in J-aggregates have been reported. However, we recently found that small π-molecules such as methyl salicylate (MS) dyad-type molecules^14^ and octahydro-binaphthols^15^ exhibit characteristic fluorescence bands in the longer-wavelength region with increasing concentration, reaching maximum intensities at significantly higher concentrations (around 0.1 M) than those at which typical fluorescent chromophores exhibit quenching. Actually, such small aromatic molecules are not expected to have effective π–π interaction,^16–18^ but we concluded that these fluorescence bands originate from π-slipped stacked aggregates formed under high concentration conditions, where intermolecular orbital mixing is compatible, resulting in delocalization of π-electrons over multiple molecules.

**Fig. 1:**
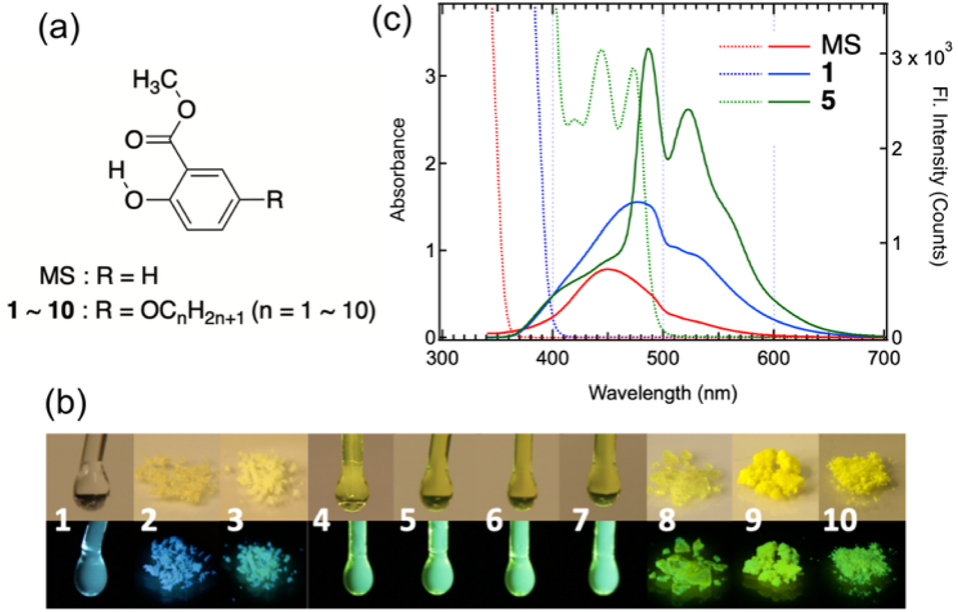
(a) Chemical structures of methyl salicylate (MS) and methyl 5-alkoxysalicylates (1–10). (b) Photographs of 1–10 taken under white light (top) and 365 nm UV light (bottom). (c) Absorption (dotted lines) and fluorescence (solid lines) spectra of neat liquid MS (red), 1 (blue), and 5 (green), measured using a 1 mm path length cell. The excitation wavelength (*λ*_ex_) was 330 nm. Fluorescence spectra of the other samples are presented in Fig. S3, SI.

Alkyl-π molecular liquids are also attracting interest as photofunctional materials,^19–29^ in which it is important to construct a concentrated system that maximizes the number of chromophores without degrading their luminescent properties.^30^ In the ongoing research on MS derivatives, we obtained highly-fluorescent molecular liquids by linking a linear alkoxy chain to MS (Fig. 1a), whose unique colour appearance and fluorescence properties cannot be explained as those of the MS molecules themselves. This suggests that microscopic local MS aggregates with π-slipped stacked structures exist within a homogeneous liquid state. Although it depends on the timescale of observation, liquids are generally isotropic and homogeneous on a macroscopic scale. Nevertheless, they often exhibit molecular-level inhomogeneity due to the formation of aggregates when effective intermolecular interactions are present.^31–34^ In particular, ionic liquids, governed by relatively strong coulombic interactions, are known to be nanoheterogeneous yet coherent and essentially regular liquids that exhibit nanoscale structural organization.^35^ Studies aiming to utilize such heterogeneity to induce the functionality of molecular liquids have been conducted, for example, on dynamic aggregates in alkyl–pyrene liquids, which give rise to excimer formation and influence liquid viscosity,^36,37^ and on insoluble aggregates formed in liquid–liquid blends that enable luminescent colour tuning.^38^ However, there have been only limited reports directly attributing the optical properties (colour and fluorescence) of molecular liquids to such multi-molecular aggregates as introduced in this study. Here, we argue that the alkoxy-chain-linked MS liquids represent a novel class of molecular liquid by confirming the validity of our interpretation.

## Results and discussion

### Optical properties of MS derivatives with varying alkoxy chain lengths

Both MS and methyl 5-methoxysalicylate (1) are colourless liquids, whereas the other synthesized samples were obtained in the following states depending on the length of the alkoxy chain (Fig. 1b, upper row): 2 and 3 were light yellow powders, 4, 5, 6, and 7 were highlighter yellow liquids, and 8, 9, and 10 were yellow powders. Although the absorption band edges of the colourless liquids (MS and 1) lie in the UV region, approximately below 400 nm (Fig. 1c, dotted red and blue lines, respectively), yellow liquids (4–7) exhibit characteristic absorption bands with fine vibronic progressions in the region of 400–500 nm (dotted green line for 5 in Fig. 1c). Similar features are also detectable when 2 or 3 are dissolved in solution at a high concentration of around 0.1 M (Fig. S1, SI). These spectral characteristics are almost the same as those observed for MS-dyads in high-concentration solutions, for which we have previously concluded that linking two MS units makes it possible to form stable aggregates with a π-stacking of MS moieties, resulting in their appearance.^14^ In this study, however, we revise our previous understanding and conclude that the stabilization of MS aggregates is achieved simply by linking alkoxy chains with two or more carbon atoms. Since all samples—including 1 to 3 as well as 4 to 10—formed colourless solutions upon dilution with solvent, giving an identical absorption spectrum with a maximum at 340 nm (Fig. S2, SI), stable π-aggregates appear to form only under condensed conditions, such as neat liquids (4–7) and highly concentrated solutions over 10^−1^ M (2, 3, 8–10).

With regard to fluorescence, both MS and 1, despite being colourless, exhibit bands in the relatively long-wavelength region of 400–600 nm (Fig. 1c, solid red and blue lines, respectively). These behaviours are due to their undergoing excited-state intramolecular proton transfer (ESIPT), which results in a large Stokes shift.^39,40^ In contrast to the blue fluorescence of MS and 1, all of the yellow liquids (4–7) exhibit light green fluorescence (Fig. 1b, lower row), whose spectra are nearly mirror images of their absorption spectra (solid green line for 5 in Fig. 1c). Even in the colourless liquid (1), however, a faint trace of this characteristic fluorescence band can be detected, when excited at 470 nm, despite the absence of absorption at that wavelength.^14^ In fact, when the yellow liquids (4–7) are sandwiched between quartz plates, they appear colourless and show no absorption in the visible region, but the characteristic fluorescence band can still be clearly observed (Fig. S4, SI). Overall, the results indicate that fluorescence spectroscopy provides a more sensitive means of detecting π-aggregates even in colourless liquids, where absorption spectroscopy yields little information, and that the π-aggregates formed in 4–7 are significantly more stable than those in 1, thereby enabling the clear expression of the characteristic absorption and fluorescence spectra.

The aggregates in the MS-liquid are considered to be passively formed as a result of molecular crowding in the highly concentrated liquid phase, in contrast to self-assembling J-aggregates of molecules possessing large association constants in solution. In practice, upon heating, no change in the spectral shape suggestive of aggregate dissociation was observed, while a decrease in intensity attributable to thermal deactivation was detected (Fig. S5, SI).

### Crystallization behaviour and thermal properties of MS liquids

**Fig. 2:**
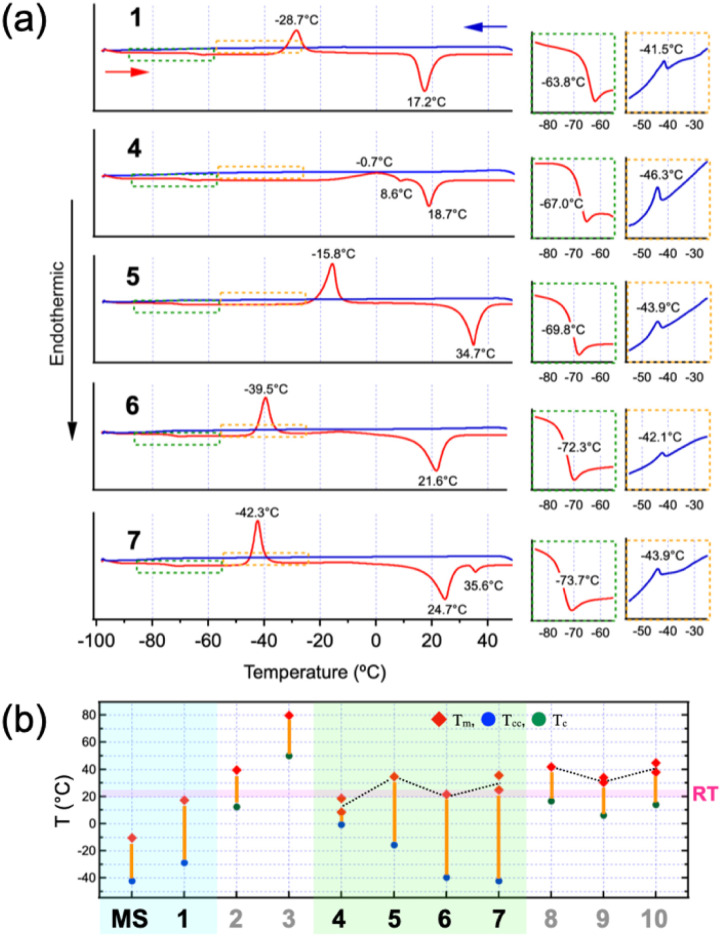
(a) DSC thermograms of liquid samples (1, 4, 5, 6, and 7). All samples were first heated from RT to 50 °C, and then subjected to cooling and reheating at a rate of 10 °C min^−1^. The cooling and heating processes are represented by blue and red lines, respectively. The regions with weak signals (highlighted by green and orange dotted lines) are enlarged and displayed on the right side. (b) Graphical representation of the thermal data presented in Table S1, SI.

To assess the thermal phase transitions of the MS liquids, differential scanning calorimetry (DSC) measurements were performed at a rate of 10 °C min^−1^ (Fig. 2a: liquid sample results; Fig. S6, SI: powder sample results; Table S1, SI: thermal data for all samples; Fig. 2b: visual representation corresponding to Table S1). Although it was hard to discern signals in the cooling traces down to −100 °C for each of the liquid samples (1, 4–7), very weak exothermic peaks were detected around −42 °C to −46 °C (orange squares with dotted outlines in Fig. 2a). On the other hand, in the subsequent heating traces, all samples exhibited very weak endothermic features, likely associated with the glass transition (*T*_g_) occurring between −74 °C and −64 °C (green squares with dotted outlines in Fig. 2a), followed by distinct exothermic crystallization peaks and endothermic melting (*T*_m_) peaks, except for 4, which exhibited a broad rise with a crystallization peak at −0.7 °C. These crystallization peaks during the heating process are attributed to crystallization from the supercooled liquid or glassy state, a phenomenon known as cold crystallization (CC). In general, CC refers to the process in which polymers, initially transformed into a glass state by cooling, undergo crystallization upon heating.^41^ However, CC has increasingly been observed even in small molecules.^25,42–46^ In the case of small molecules, glass formation, supercooling, and crystallization compete with one another, influenced by factors such as diffusion and crystallization rates, molecular geometry and flexibility and intermolecular interactions; in fact, the rapid glass-to-crystal (GC) transition occurring near *T*_g_ of fragile liquids has been recognized as another key phenomenon in the thermal phase transitions of small molecules.^47–51^ Among the molecules exhibiting CC reported so far, the MS-based liquid is, to our knowledge, the smallest, comparable in size to the imidazolium-based ionic liquids.^52^ The CC behaviour observed in the MS liquids is likely not explained solely by van der Waals (vdW) interactions between the alkoxy chains, since alkanes such as butane, pentane, hexane, and heptane all exhibit much lower freezing points (below approximately −100 °C). Thus, the occurrence of CC implies that the MS π-moiety has a tendency to aggregate, even though it possesses only a small π-system and lacks coulombic interactions typically observed in the imidazolium-based ionic liquids. Based on the CC temperatures (*T*_cc_) of the yellow liquids, their crystallizability increases with alkoxy chain length (7 > 6 > 5 > 4), suggesting that alkoxy chain interactions may support crystallization alongside the primary stacking of the MS π-moieties. Notably, even MS itself exhibits CC (Fig. S7, SI). However, the stacking structures of MS and 1, both of which are colourless liquids, must differ from those of the yellow liquids (4–7). This suggests that the aggregates responsible for the yellow coloration are not efficiently formed if alkoxy chain interactions are absent. On the other hand, their *T*_m_ values, which are close to room temperature (RT), show a slight indication of an odd–even effect (Fig. 2b). As an additional note, the appearance of two *T*_m_ peaks in 4 and 7 may suggest a possibility that their crystalline states are polymorphic.

### X-ray diffraction analysis of MS liquids

**Fig. 3:**
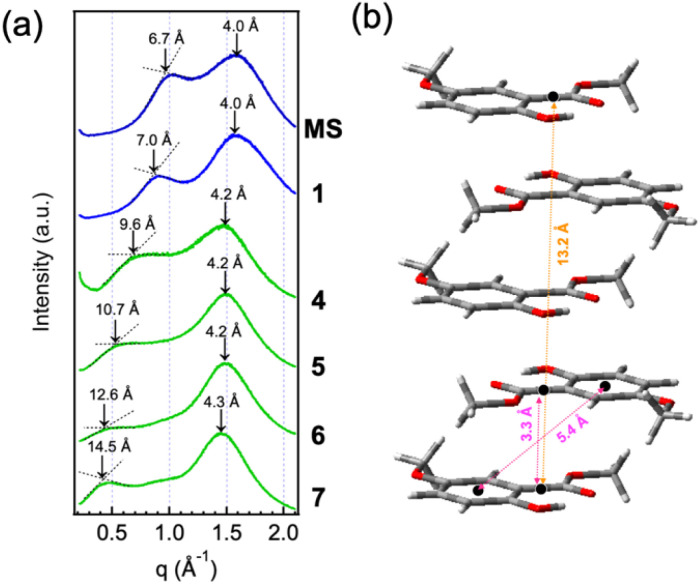
(a) XRD patterns of liquid samples (MS, 1, 4, 5, 6, and 7) at RT. (b) The pentamer model for MS aggregates in the liquid state, constructed using five molecules of 1 as described in ref. 14.

To confirm the presence of microscopic local π-aggregate structures within the macroscopically homogeneous liquid phase, X-ray diffraction (XRD) measurements were carried out. As a result, two halos were detected (Fig. 3a). The left-side halos shift to lower *q* values as the alkoxy chain length increases, whereas the right-side halos appear around *q* ≈ 1.5 Å^−1^, irrespective of the chain length. In fact, alkyl-π molecular liquids often exhibit similar XRD profiles with two such halos: one originating from molten aliphatic chains, and the other from the averaged distance between π units.^9,25,53–55^ Compared to these alkyl-π molecules, however, our MS molecules are much more compact in size, both in their π and chain moieties, and not bulky. For this reason, similar attributions would not be suitable. The *d*-spacing of 4.0–4.3 Å, obtained from *q* ≈ 1.5 Å^−1^, is approximately in agreement with that of the benzene dimer in the offset parallel stacking mode, as determined from neutron diffraction results for liquid benzene.^56,57^ We previously reported the tentative model of the MS aggregates using methyl 5-methoxysalicylate (*i.e.*, 1) molecules, where they adopt a slipped antiparallel stacking arrangement. In this model (Fig. 3b), the interplanar distance is 3.3 Å, and the center-to-center distance between the aromatic rings is 5.4 Å. Considering that, on the time scale of XRD measurements, the molecules are sufficiently dynamic and are not fixed in static local structures, it is reasonable to assume that this dimeric configuration could give rise to a diffraction halo corresponding to a *d*-spacing of approximately 4.2 Å. On the other hand, the left-side halos, which shift depending on the chain length, give much larger *d*-spacing values. Since it is impossible to assume these large distances using an MS molecule with a single benzene ring, π-aggregates involving more than a dimer must be considered. Based on the combined interplanar distances in the aggregation model, π-aggregates ranging from trimers to pentamers could exist within the macroscopically homogeneous liquid phase. In addition, elongation of the alkoxy chain appears to promote aggregate growth, enabling the formation of larger aggregates. It is experimentally difficult to determine the molecular arrangement within aggregates in the neat liquid state. Therefore, molecular dynamics (MD) simulations remain a future challenge for us.

### Liquid physical properties related to molecular mobility

**Fig. 4:**
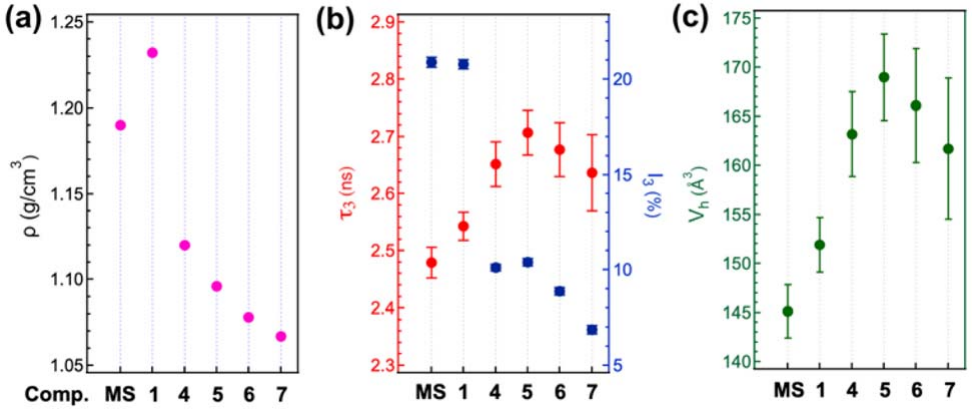
Density and free volume of the MS-based RT liquids plotted against chain length: (a) density (*ρ*); (b) *τ*_3_ and *I*_3_ values, represented by red and blue markers, respectively, and obtained from positron annihilation lifetime spectroscopy (PALS); (c) hole volume (*V*_h_) estimated from *τ*_3_ using Tao–Eldrup model. See Table S2 for details of the data and calculations.

Although the viscosity of the MS liquids was not experimentally measured, it is presumed to be low and comparable to that of common organic solvents, as suggested by its flow behaviour during handling (see Movie S1, SI). Such fluidity appears reasonable, given the small and simple structure of the MS molecules and their weak intermolecular interactions. With regard to density (*ρ*), there is a distinct discontinuity between the colourless liquids (MS and 1) and the yellow liquids (4–7), and a gradual decrease is observed as the alkoxy chain length increases from 4 to 7 (Fig. 4a). On the other hand, free volume is a key parameter for understanding molecular mobility and viscosity, and was therefore also evaluated using positron annihilation lifetime spectroscopy (PALS) (Fig. S8 and Table S2, SI). In this technique, the longest-lived component of the positron lifetime spectrum (*τ*_3_) is attributed to *ortho*-positronium annihilation in intermolecular free-volume regions, and its intensity (*I*_3_) reflects the relative amount of such free volume.^54^ In the present system, the *τ*_3_ values are approximately in the range of 2.5 to 2.7 ns, irrespective of chain length (Fig. 4b, red makers). Based on the Tao–Eldrup model,^58,59^ which approximates the free-volume holes as spheres, the mean hole radius can be estimated from *τ*_3_, corresponding to a hole volume (*V*_h_) in the range of 140 to 170 Å^3^ (Fig. 4c). Although this model provides a static estimate of free volume, the values could be used as a structural parameter to discuss trends in dynamic free volume in soft matter systems.^60^ The larger *V*_h_ observed in the yellow liquids (4–7), compared to the colourless liquids (MS and 1), may be due to the fact that longer alkoxy chains make molecular packing less efficient, resulting in larger free volume. However, when comparing among the yellow liquids (4–7), the gradual decrease in *V*_h_ with increasing chain length may suggest that longer chains allow for more efficient molecular packing, *i.e.*, the formation of π-aggregates; such an interpretation is also consistent with the DSC and XRD results mentioned above. On the other hand, it should be noted that the *I*_3_ value for the yellow liquids is markedly lower (approximately 10% or less), indicating fewer free-volume regions—less than half of that observed for the colourless liquids (Fig. 4b, blue makers). This further supports the interpretation that the MS moieties in the yellow liquids are closely stacked, aided by the vdW interactions between the alkoxy chains.

### Fluorescence lifetime measurement of the MS samples

**Fig. 5:**
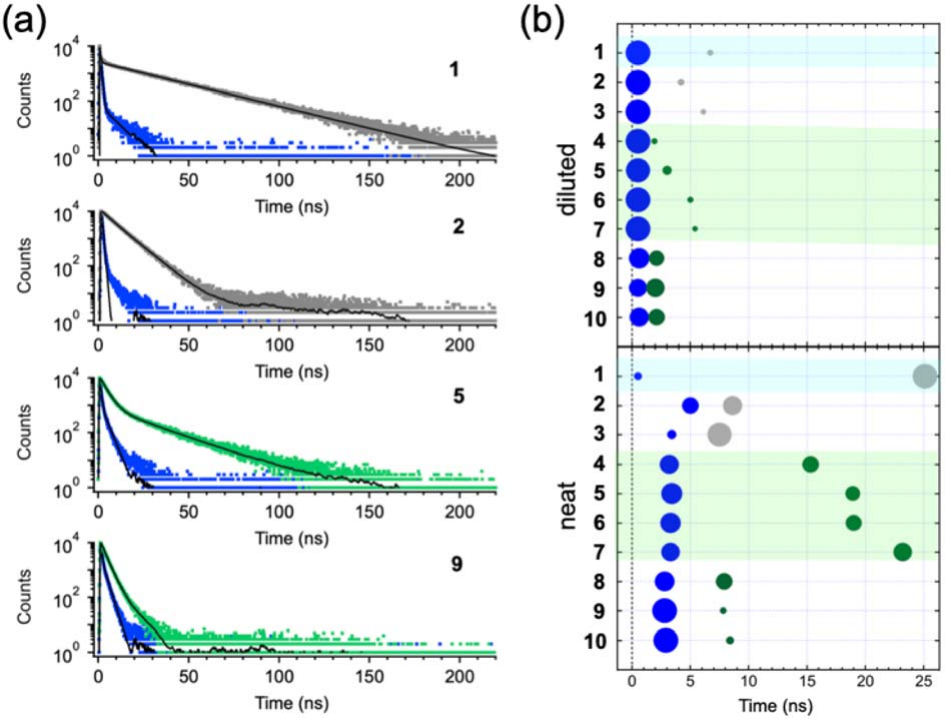
(a) Time-resolved fluorescence decay curves of liquid samples 1 and 5, and powder samples 2 and 9. Blue traces correspond to diluted samples (3 × 10^−4^ M in chloroform; *λ*_ex_ = 375 nm, *λ*_em_ = 400 nm). Grey and green traces represent neat samples (*λ*_ex_ = 375 nm, *λ*_em_ = 450 nm for 1; 400 nm for 2 and 3; 500 nm for 4–10). (b) Visual representation of the numerical data presented in Table S3, SI. Blue and green circles indicate the short- and long-lifetime components, respectively, with circle size reflecting their relative contributions.

To verify that the characteristic fluorescence of the MS liquid arises from the formation of supramolecular π-aggregates, time-resolved fluorescence spectroscopy measurements were conducted (Fig. 5a: representative sample results; Table S3, SI: analytical data for all samples; Fig. 5b: visual representation corresponding to Table S3). Regardless of whether the sample is a powder (*e.g.*, 2 and 9) or a liquid (*e.g.*, 1 and 5), their dilute solutions exhibited identical fluorescence spectra (Fig. S1, SI) and nearly overlapping fluorescence decay curves, with a lifetime (*τ*) of 0.5 ns (Fig. 5a, blue plots), although additional components with *τ* values approximately an order of magnitude longer were moderately observed in samples 8, 9, and 10 (Fig. 5b, top).

On the other hand, the decay curves for the neat samples are significantly broader than those for their dilute solutions (Fig. 5a, grey and green plots). In particular, this broadening is noticeable in the liquid samples of 1 and 5, for which exponential fittings indicate the presence of two components: a fast *τ*_1_ of a few ns and a slower *τ*_2_ of approximately 20 ns (Fig. 5b, bottom). In 1, the slower *τ*_2_ component accounts for nearly 90% of the total emission, indicating that most of the excitation energy is received by π-aggregates that are too unstable to exhibit the characteristic fluorescence observed in the yellow liquids (4–7). The dominant *τ*_2_ component thus likely originates from these weakly bound π-aggregates. In contrast, in the yellow liquids, the *τ*_2_ components constitute 30–50% of the total emission, increasing with chain elongation. Since the π-aggregates in the yellow liquids are not effectively excited at the excitation wavelength *λ*_ex_ = 375 nm as their absorption band is located in the 400–500 nm region, it is plausible that the fluorescence observed at *λ*_em_ = 500 nm is caused by energy transfer from monomers (faster *τ*_1_ component) to the π-aggregates (slower *τ*_2_ component). Considering this, the presence of these two *τ* components clearly demonstrates the coexistence of monomers and π-aggregates in the yellow liquids. It is likely that, on the timescale of fluorescence, the π-aggregates in 4–7 are tightly bound enough to enable delocalized excitation over multiple molecules, giving rise to their characteristic optical properties. Furthermore, it is evident that such π-aggregates, associated with the slower *τ*_2_ component, are not present in the yellow powder samples 8–10.

### Enhanced π-aggregate formation in the melted state

**Fig. 6:**
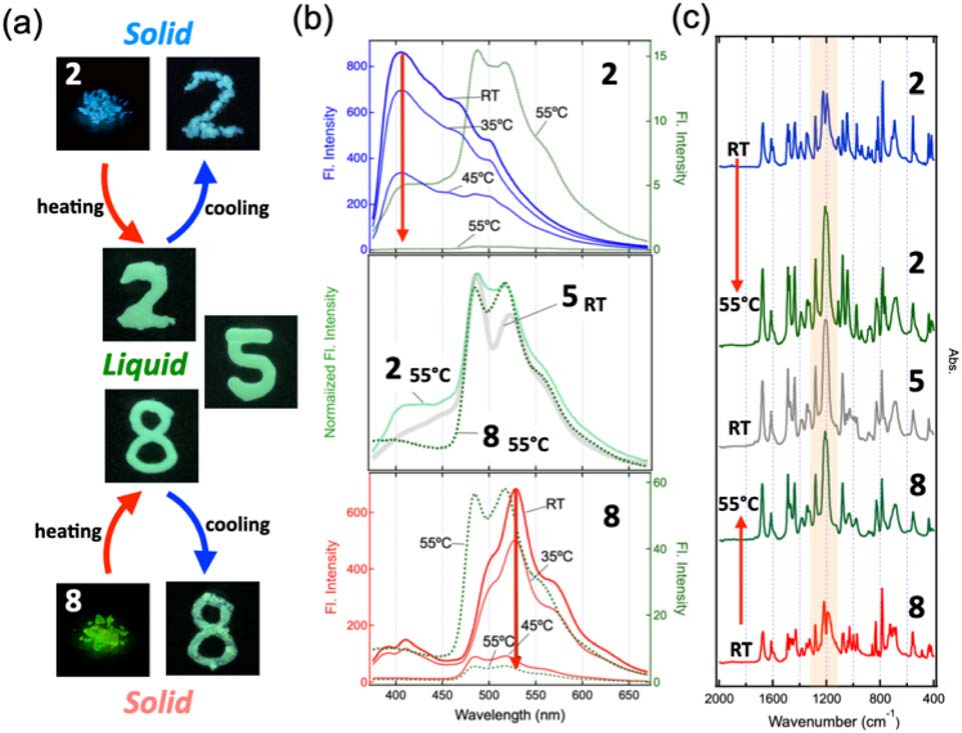
(a) Photographs of 2 and 8 under 365 nm UV irradiation: before and after heating at 55 °C, and after cooling, along with the RT liquid 5. (b) Fluorescence spectral changes of 2 (top) and 8 (bottom) upon heating. The middle panel shows a spectral comparison of 2 and 8 at 55 °C with 5. (c) IR spectral changes of 2 and 8 upon heating to 55 °C, compared with the spectrum of 5 at RT. IR spectra of the other samples at RT are provided in Fig. S9, SI.

According to the DSC results (Fig. 2b), all powders except for 3 melted at relatively low temperatures below 50 °C and formed a liquid phase. These liquids became capable of exhibiting light green fluorescence, similar to that of the RT liquids of 4–7. Notably, even 2 and 3, which were originally blue fluorescent powders, turned into light green fluorescent liquids upon melting (Fig. 6a). For both 2 and 8, although the fluorescence intensities decreased markedly upon heating, the melted states exhibited the same fluorescence spectra as the RT liquid of 5, in which the two vibronic peaks at 487 nm (20 533 cm^−1^) and 518 nm (19 305 cm^−1^) exactly matched (Fig. 6b, mid panel). This indicates that the fluidity caused by melting promotes the formation of π-aggregates, which appears to be identical to those present in the RT liquids. In contrast, the crystalline phase of the RT liquid 4, obtained by cold crystallization near 0 °C, exhibited a fluorescence spectrum almost identical to that of 8 (Fig. S10, SI). Therefore, π-aggregates are observed exclusively in the liquid state.

The π-aggregate formation resulting from heating and dissolving the powder samples induces a distinct IR spectral change, with the signal around 1200 cm^−1^ being significantly enhanced (Fig. 6c). We previously investigated the origin of the fine vibronic progression observed in the absorption and fluorescence spectra of MS-dyads by carrying out a normal-mode analysis of the π-aggregate model shown in Fig. 3b.^14^ The results suggested that association of the MS molecules activates a vibrational mode mainly characterized by the C–O stretching of the methylcarboxyl group, which evolves into a collective motion involving all constituent MS molecules of the π-aggregate. We thus concluded that such a collective mode is strongly coupled with the electronic transition of the π-aggregate, thereby giving rise to the fine vibronic progression of the spectra. When this interpretation is applied to the present system, the energy of the association-induced collective mode is about 1200 cm^−1^, which is in reasonable agreement with the energy difference between the two vibronic peaks (1228 cm^−1^). Therefore, the IR measurements of the neat sample in this work successfully captured the vibrational states before and after aggregation; as a result, our interpretation of the origin of the vibronic progression was experimentally verified.

Moreover, XRD measurements were performed on the heated sample to detect possible π-aggregates in the melted state. The sample of 9 was measured immediately after heating and melting. Although crystallization progressed over time as the sample cooled down resulting in several sharp peaks, a halo with a *d*-spacing of 19.3 Å, larger than that of 7 shown in Fig. 3a, was also observed (Fig. S11, SI). The above results demonstrate that MS tends to form π-aggregates only in the condensed liquid phase when its alkoxy chain contains two or more carbon atoms, and that the extent of aggregate growth increases with the alkoxy chain length.

## Conclusions

In conclusion, we demonstrated that alkoxylation of methyl salicylate (MS) at the 5-position induces the formation of supramolecular π-aggregates in the liquid state, significantly altering its optical properties. The transformation to a vivid yellow liquid with distinct absorption and fluorescence features was observed for derivatives bearing alkoxy chains of four or more carbon atoms (4–7), suggesting extended π-electron delocalization across multiple MS units. Structural characterization based on XRD measurements confirmed the presence of π-aggregates with dimensions of 10–15 Å, involving three to five MS molecules arranged *via* face-to-face π-stacking. Supporting this aggregation behaviour, DSC measurements suggested that cold crystallization originates from π-aggregation under high-density conditions, despite the small and simple molecular structure. PALS measurements further suggested that the yellow liquids (4–7) are in a more densely packed state compared to the colourless liquids (MS and 1). In addition, fluorescence lifetime measurement of 4–7 revealed a long-lived emission component arising from energy transfer from the monomeric state to the π-aggregated state. These results provide direct evidence that even relatively simple and small molecules can lead to microscopic local structural aggregates in liquids, with substantial influence on macroscopic properties such as colour and fluorescence. These results provide direct evidence that even relatively simple and small molecules can lead to microscopic local structural aggregates in liquids, with substantial influence on macroscopic properties such as colour and fluorescence. Only weak intermolecular interactions—such as dipole–dipole and dispersion forces between the small π-systems of the constituent MS molecules—are present within these aggregates. This is in contrast to ionic liquids, where structural heterogeneity is formed by the distinct coulombic attraction between the positively and negatively charged components, as well as by the possible formation of hydrogen-bonding networks.^35^ Nevertheless, π-aggregates with delocalized π-electrons across multiple molecules may form due to several factors, including (1) the realization of a condensed state unique to liquids, (2) compatibility of intermolecular orbital mixing,^14^ and (3) the presence of alkyl chains of appropriate length that stabilize the π-stacking structure. Following these design principles, we have tentatively identified additional potential candidates besides the MS liquids. The present study demonstrates that the condensed state of liquids uniquely enables the formation of π-aggregates capable of generating new electronic states, offering a promising platform for the development of advanced optical and electronic functionalities.

## Author contributions

K. K. carried out a series of measurements. K. S. conceived and supervised the project and wrote the manuscript. T. A. and T. N. also supervised the project.

## Conflicts of interest

There are no conflicts to declare.

## Supplementary Material

SC-OLF-D5SC06148B-s001

SC-OLF-D5SC06148B-s002

## Data Availability

All data supporting the findings of this study are included within the article and its supplementary information (SI) files. Supplementary information: experimental details, synthetic procedures, and additional data such as optical, DSC, PALS, fluorescence lifetime, ATR-IR, and XRD measurements (Fig. S1–S11 and Tables S1–S3). See DOI: https://doi.org/10.1039/d5sc06148b.
