# Supplementary material for: Harnessing inhomogeneous π-aggregates: a new path to optical modulation in methyl salicylate-based solvent-free liquids
Source: Chem Sci. 2025 Oct 22;16(46):21797–805. doi: 10.1039/d5sc06148b (PMC12560049; doi:10.1039/d5sc06148b)
Supplement: SC-016-D5SC06148B-s002 [file SC-016-D5SC06148B-s002.pdf]

Supplementary Information (SI) for Chemical Science.
This journal is © The Royal Society of Chemistry 2025

Supplementary information

Harnessing inhomogeneous π-aggregates: A new path to optical modulation in methyl salicylate-based solvent-free liquids

*Kei Kobayashi,[a] Ken-ichi Sakai *[a] Tomoyuki Akutagawa[b] and Takashi Nakanishi [c]*

[a]Department of Applied Chemistry and Bioscience, Chitose Institute of Science and Technology (CIST), Chitose 066-8655, Japan

[b]Polymer Hybrid Materials Research Center, Institute of Multidisciplinary Research for Advanced Materials (IMRAM), Tohoku University, Sendai 980-8577, Japan

[c]Research Center for Materials Nanoarchitectonics (MANA), National Institute for Materials Science (NIMS), 1-1 Namiki, Tsukuba 305-0044, Japan.

----- Contents -----

Experimental Section

1. Measurements
2. Synthesis and characterization of methyl salicylate (MS) derivatives **2–10**

・Sample preparation

・Analysis by electron-impact ionization mass spectrometry

・Identification by ^{1}H and ^{13}C NMR spectroscopy

Supplementary Figures and Tables

1. Additional data related to optical measurements (**Figs. S1–S5)**
2. Supplementary data from DSC measurements (**Figs. S6** and **S7**; **Table S1)**
3. PALS measurements for liquid samples of **MS**, **1**, and **4–7** (**Fig. S8**; **Table S2)**
4. Summary of fluorescence lifetime measurements (**Table S3)**
5. ATR-IR spectra (**Fig. S9)**
6. Fluorescence spectrum of **4** after cold crystallization (**Fig. S10)**
7. XRD pattern of **9**, recorded immediately after thermal dissolution (**Fig. S11)**

References

Experimental Section

1. Measurements

The instruments used for measurements conducted in this study are listed below.

- **NMR spectroscopy:** AVANCE NEO 400 MHz spectrometer (Bruker)
- **Mass spectrometry:** JMS-Q1050GC mass spectrometer with a direct injection probe (JEOL)
- **Absorption spectroscopy**: UV-2500PC spectrometer (Shimadzu)
- **Fluorescence spectroscopy**: FP-8300 spectrofluorometer (JASCO)
- **Temperature-controlled fluorescence measurements**: CoolSpek USP-203 (UNISOKU)
- **Differential scanning calorimetry (DSC):** DSC 7000X (Hitachi High-Tech Science), equipped with liquid nitrogen or an electronic cooling accessory, operated under nitrogen flow.
- **X-ray diffraction (XRD) measurements:** MiniFlex 600-C (Rigaku) using Cu Kα radiation (λ = 1.5418 Å).
- **Fourier-transform infrared (FT-IR) spectroscopy:** IRXross FT-IR spectrophotometer (Shimadzu) equipped with a GradiATR attenuated total reflectance (ATR) accessory (PIKE Technologies).
- **Nanosecond time-resolved fluorescence lifetime measurements:** FluoroCube 3000U-UltraFast-SP spectrophotometer, equipped with a nanosecond pulse LED (PB-373, 279 nm) and a nanosecond photodetection module (TBX).
- **Positron annihilation lifetime spectroscopy (PALS)**: Positron surface analyzer (PSA) type L-II (TOYOSEIKO)
- **Density**: Micromeritics AccuPyc II 1340, using 1.0 cm^3 inner cell (Shimadzu)

2. Synthesis and characterization

・**Sample preparation**: Methyl salicylate (MS) and methyl 5-methoxysalicylate (**1**) were purchased from Tokyo Chemical Industry Co., Ltd. (TCI) and used without further purification. The other alkoxylated MS derivatives (**2–10**) were synthesized by referring to previously reported methods.[1] Synthesis of **5**: Methyl 2,5-dihydroxybenzoate (5.0 g, 29.8 mmol), 1-bromopentane (4.5 g, 29.8 mmol), and sodium carbonate (7.4 g) were dissolved in dry acetone (250 mL) and stirred at 50 °C overnight. After evaporation, the reaction residue was dissolved in CH_2Cl_2 (300 mL), washed with water (3 × 100 mL), and dried over anhydrous $MgSO_4$. The solvent was removed, and the residue was purified by short column chromatography on silica gel (CH_2Cl_2) to afford compound **5** as a yellow oil (2.6 g, 37%). Other derivatives were also synthesized in a similar manner using the corresponding bromoalkane and were obtained in moderate yields.

・**Analysis by electron-impact ionization mass spectrometry**

2 (n = 2) $C_{10}H_{12}O_4$ f. w. = 196.2

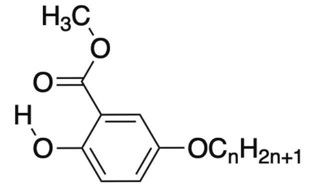

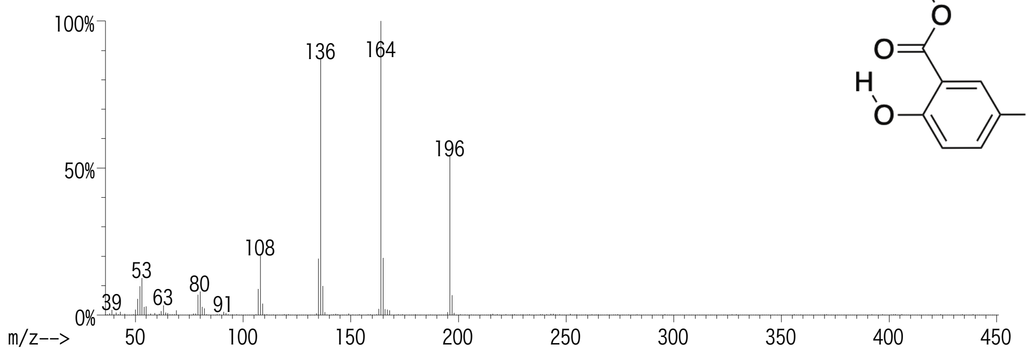

3 (n = 3) $C_{11}H_{14}O_4$ f. w. = 210.2

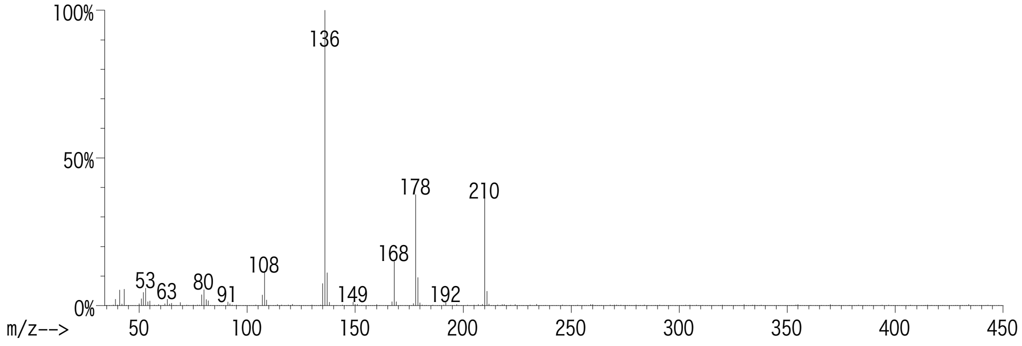

4 (n = 4) $C_{12}H_{16}O_4$ f. w. = 224.3

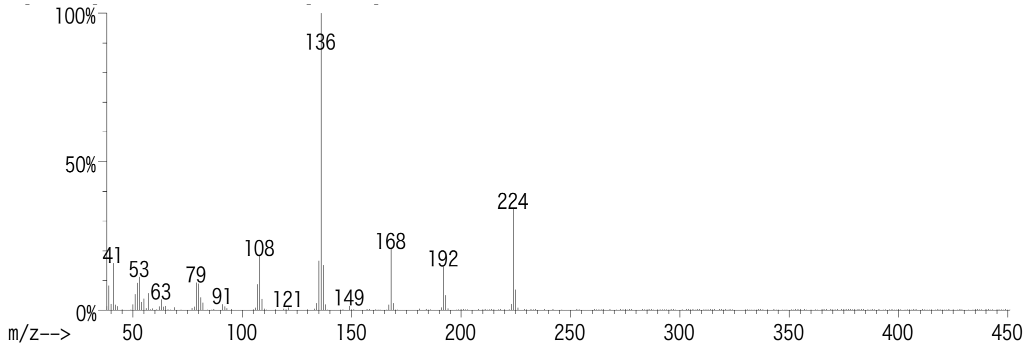

5 (n = 5) $C_{13}H_{18}O_4$ f. w. = 238.3

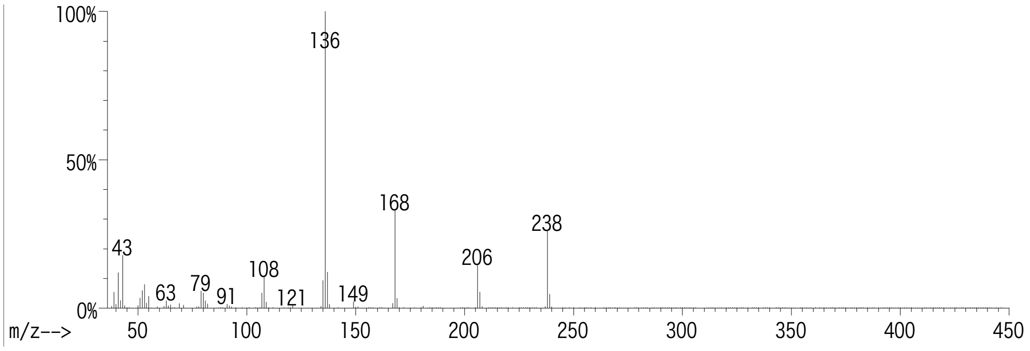

6 (n = 6) $C_{14}H_{20}O_4$ f. w. = 252.3

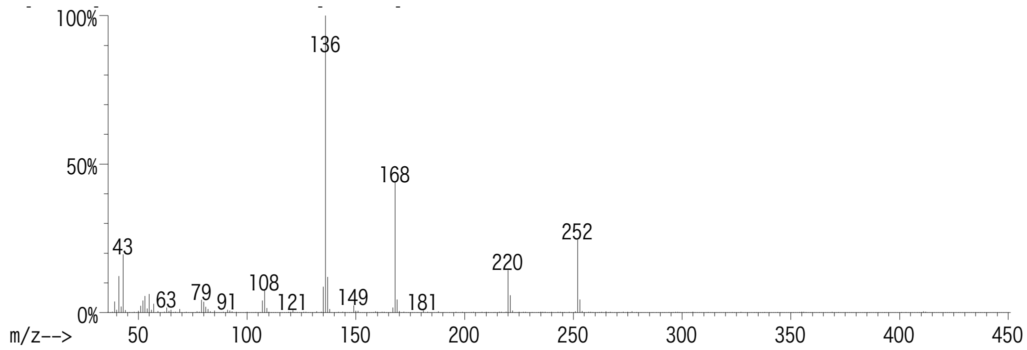

7 (n = 7) $C_{15}H_{22}O_4$ f. w. = 266.3

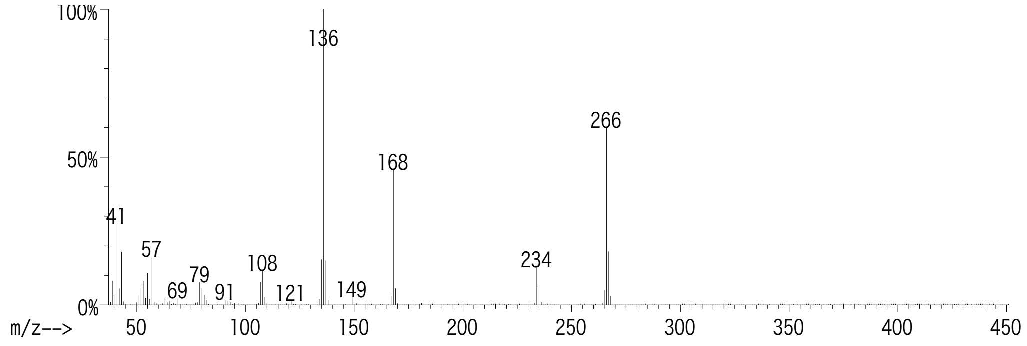

8 (n = 8) $C_{16}H_{24}O_4$ f. w. = 280.3

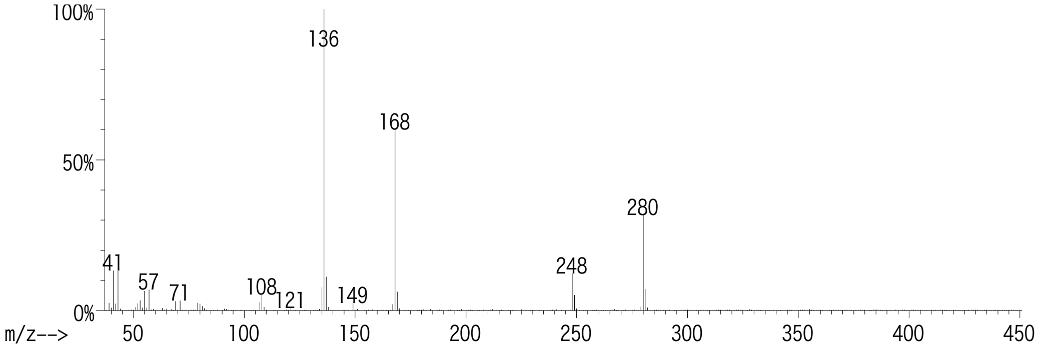

9 (n = 9) $C_{17}H_{26}O_4$ f. w. = 294.4

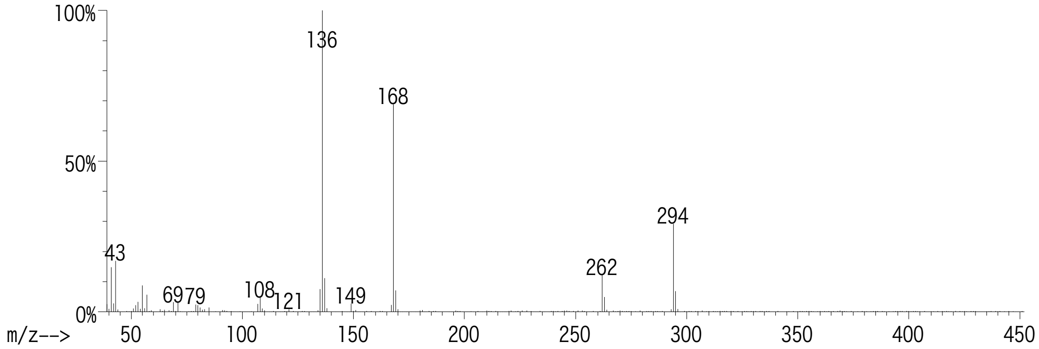

10 (n = 10) $C_{18}H_{28}O_4$ f. w. = 308.4

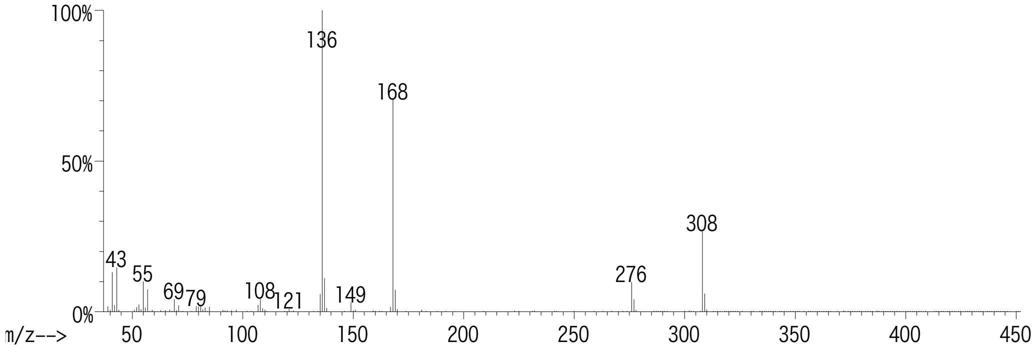

・Identification by ^{1}H and ^{13}C NMR Spectroscopy

Methyl 5-ethoxy-2-hydroxybenzoate (2)

^{1}H NMR (400 MHz, CHLOROFORM-*d*) δ ppm 1.40 (t, *J*=7.00 Hz, 3 H) 3.95 (s, 3 H) 3.99 (q, *J*=7.00 Hz, 2 H) 6.91 (d, *J*=9.01 Hz, 1 H) 7.08 (dd, *J*=9.01, 3.13 Hz, 1 H) 7.29 (d, *J*=3.13 Hz, 1 H) 10.35 (s, 1 H)

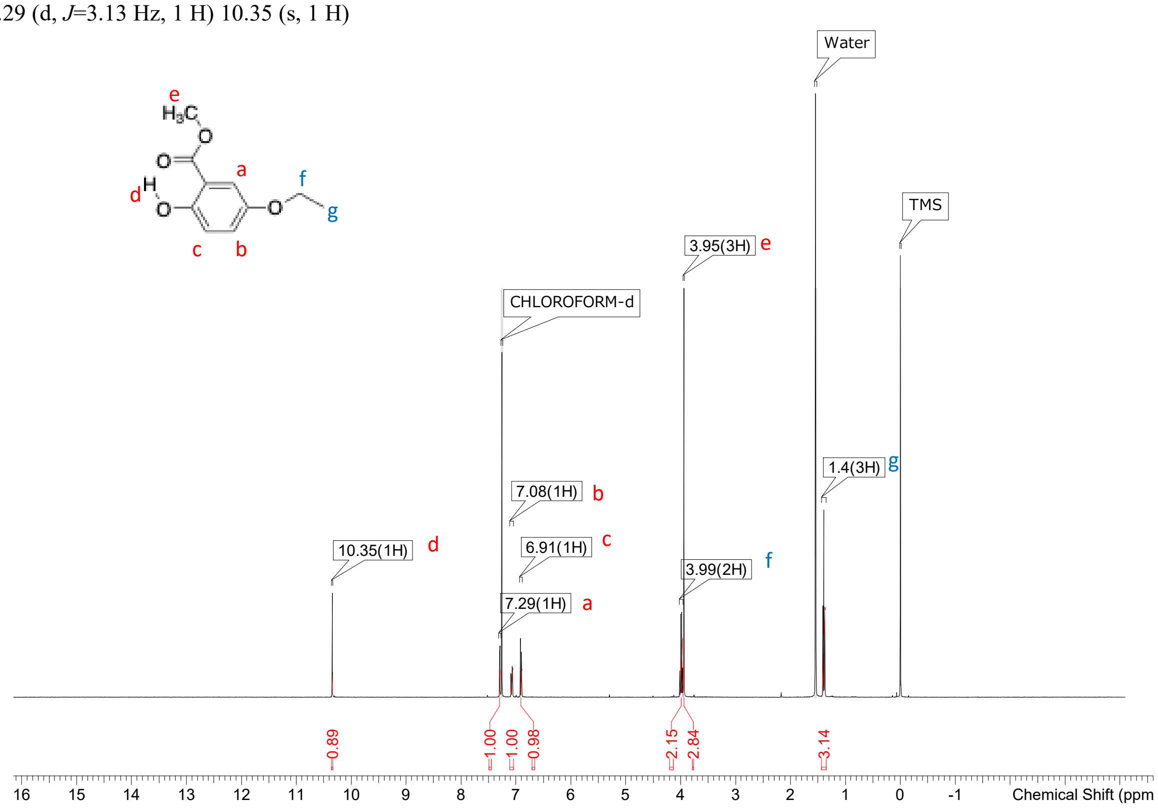

^{13}C NMR (100 MHz, CHLOROFORM-*d*) δ ppm 14.8 (s, 1 C) 52.3 (s, 1 C) 64.3 (s, 1 C) 111.7 (s, 1 C) 112.9 (s, 1 C) 118.5 (s, 1 C) 124.6 (s, 1 C) 151.3 (s, 1 C) 156.0 (s, 1 C) 170.3 (s, 1 C)

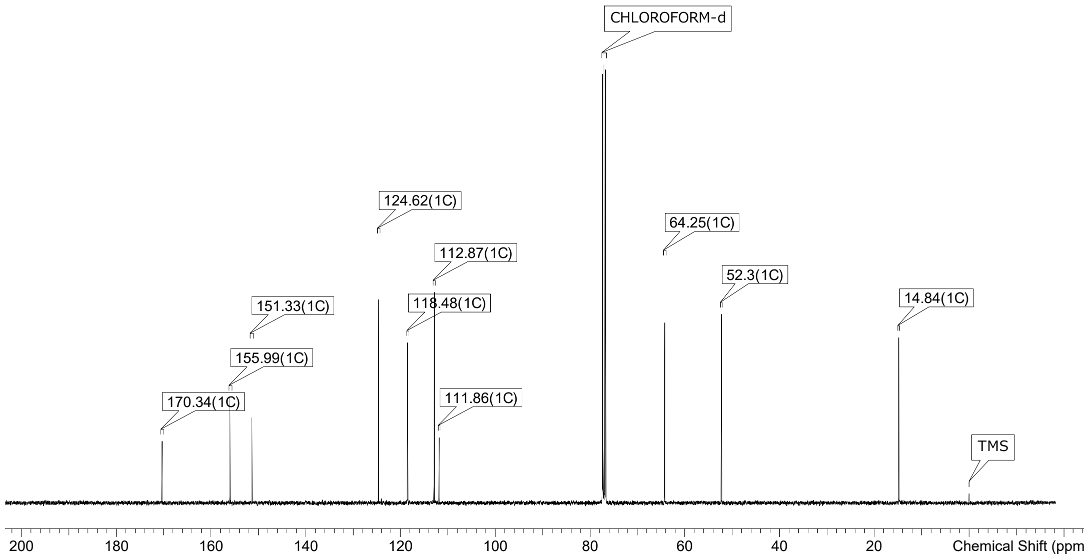

Methyl 2-hydroxy-5-propoxybenzoate (3)

^{1}H NMR (400 MHz, CHLOROFORM-*d*) δ ppm 1.03 (t, *J*=7.44 Hz, 3 H) 1.67 - 1.89 (m, 2 H) 3.88 (t, *J*=6.57 Hz, 2 H) 3.95 (s, 3 H) 6.91 (d, *J*=9.01 Hz, 1 H) 7.07 (d, *J*=3.13 Hz, 1 H) 7.29 (d, *J*=3.13 Hz, 1 H) 10.34 (s, 1 H)

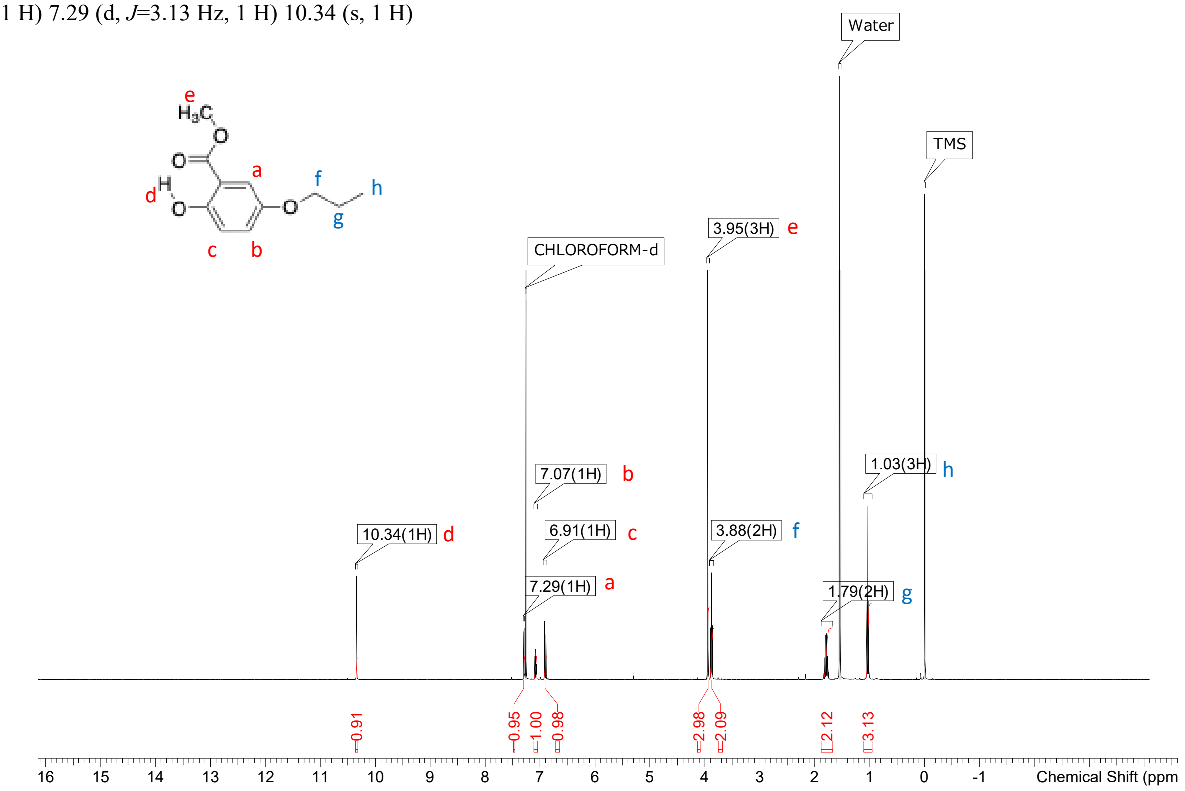

^{13}C NMR (100 MHz, CHLOROFORM-*d*) δ ppm 10.5 (s, 1 C) 22.6 (s, 1 C) 52.3 (s, 1 C) 70.3 (s, 1 C) 111.8 (s, 1 C) 112.8 (s, 1 C) 118.4 (s, 1 C) 124.6 (s, 1 C) 151.6 (s, 1 C) 155.9 (s, 1 C) 170.4 (s, 1 C)

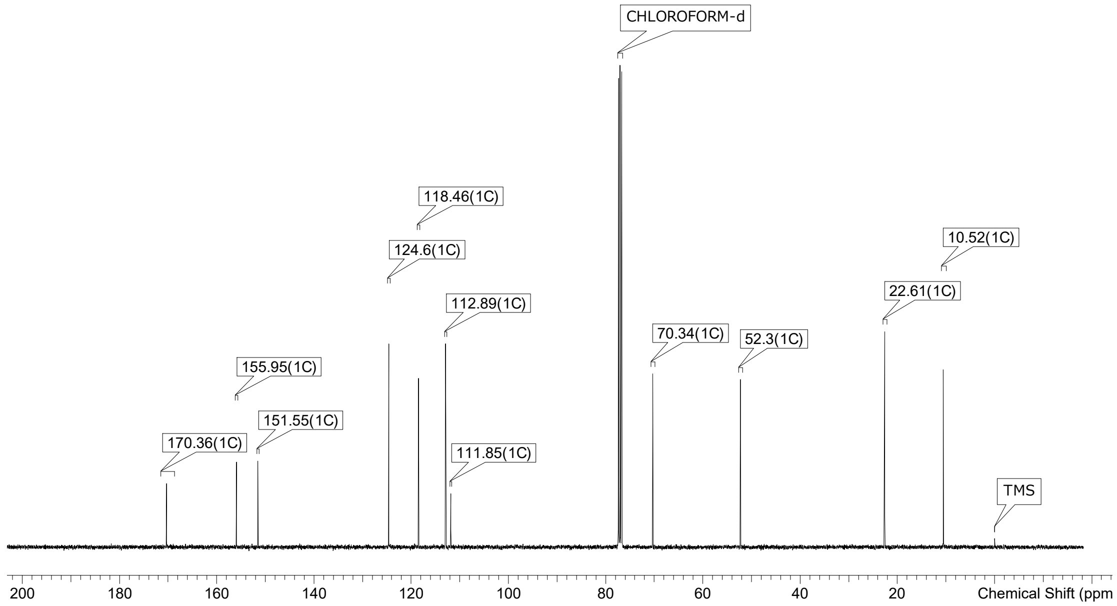

Methyl 5-butoxy-2-hydroxybenzoate (4)

^{1}H NMR (400 MHz, CHLOROFORM-*d*) δ ppm 0.98 (t, *J*=7.38 Hz, 3 H) 1.44 - 1.54 (m, 2 H) 1.71 - 1.79 (m, 2 H) 3.92 (t, *J*=6.50 Hz, 2 H) 3.95 (s, 3 H) 6.91 (d, *J*=9.13 Hz, 1 H) 7.08 (dd, *J*=9.01, 3.13 Hz, 1 H) 7.29 (d, *J*=3.19 Hz, 1 H) 10.34 (s, 1 H)

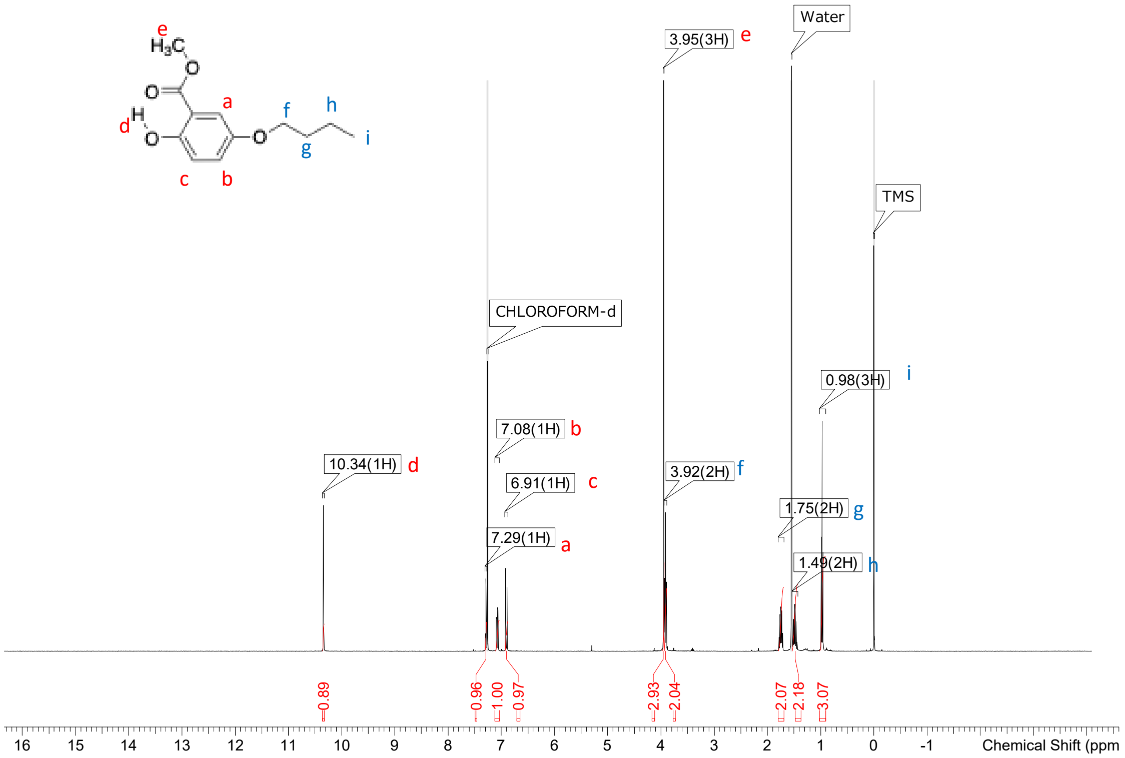

^{13}C NMR (100 MHz, CHLOROFORM-*d*) δ ppm 13.9 (s, 1 C) 19.2 (s, 1 C) 31.4 (s, 1 C) 52.3 (s, 1 C) 68.5 (s, 1 C) 111.8 (s, 1 C) 112.8 (s, 1 C) 118.4 (s, 1 C) 124.6 (s, 1 C) 151.6 (s, 1 C) 155.9 (s, 1 C) 170.4 (s, 1 C)

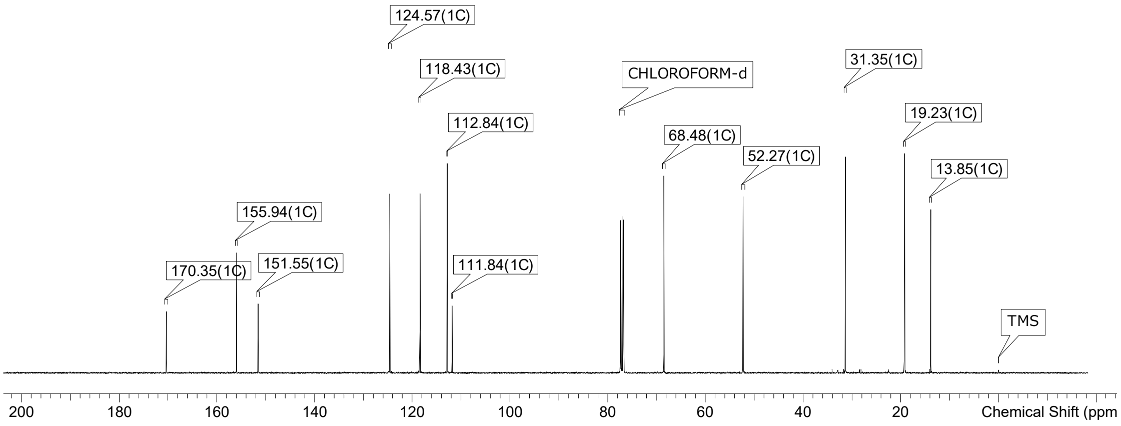

Methyl 2-hydroxy-5-(pentyloxy)benzoate (5)

^{1}H NMR (400 MHz, CHLOROFORM-*d*) δ ppm 0.89 - 0.97 (m, 3 H) 1.32 - 1.49 (m, 4 H) 1.71 - 1.82 (m, 2 H) 3.91 (t, *J*=6.57 Hz, 2 H) 3.95 (s, 3 H) 6.91 (d, *J*=9.01 Hz, 1 H) 7.08 (dd, *J*=9.01, 3.13 Hz, 1 H) 7.29 (d, *J*=3.13 Hz, 1 H) 10.34 (s, 1 H)

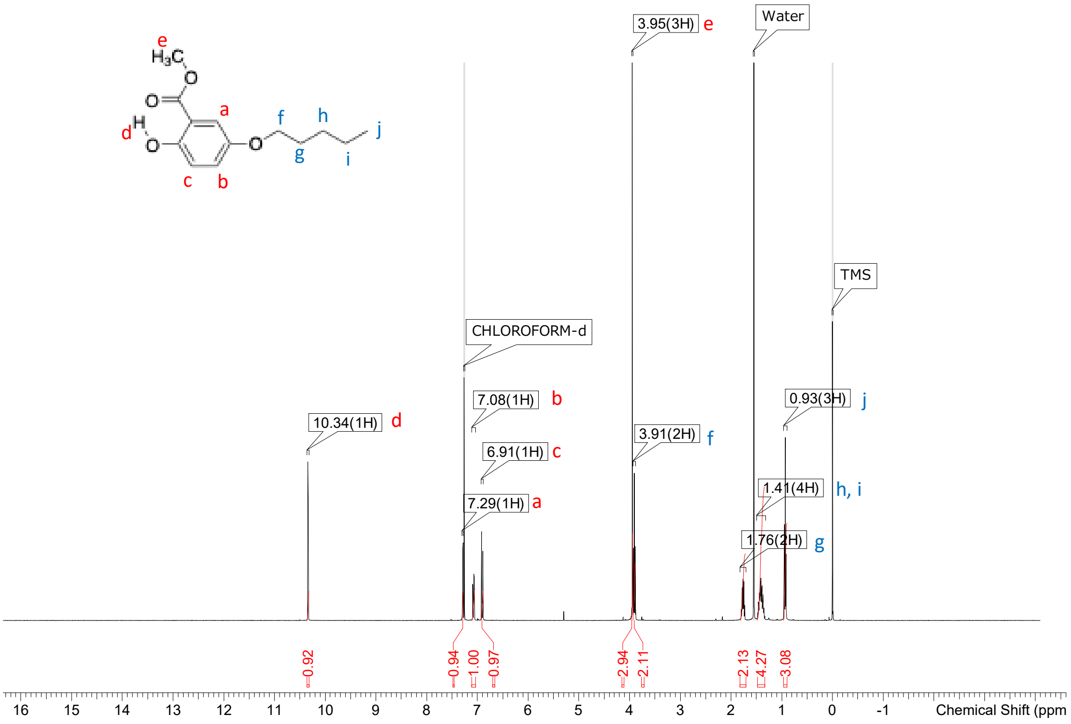

^{13}C NMR (100 MHz, CHLOROFORM-*d*) δ ppm 14.0 (s, 1 C) 22.5 (s, 1 C) 28.2 (s, 1 C) 29.0 (s, 1 C) 52.3 (s, 1 C) 68.8 (s, 1 C) 111.9 (s, 1 C) 112.8 (s, 1 C) 118.4 (s, 1 C) 124.6 (s, 1 C) 151.6 (s, 1 C) 155.9 (s, 1 C) 170.4 (s, 1 C)

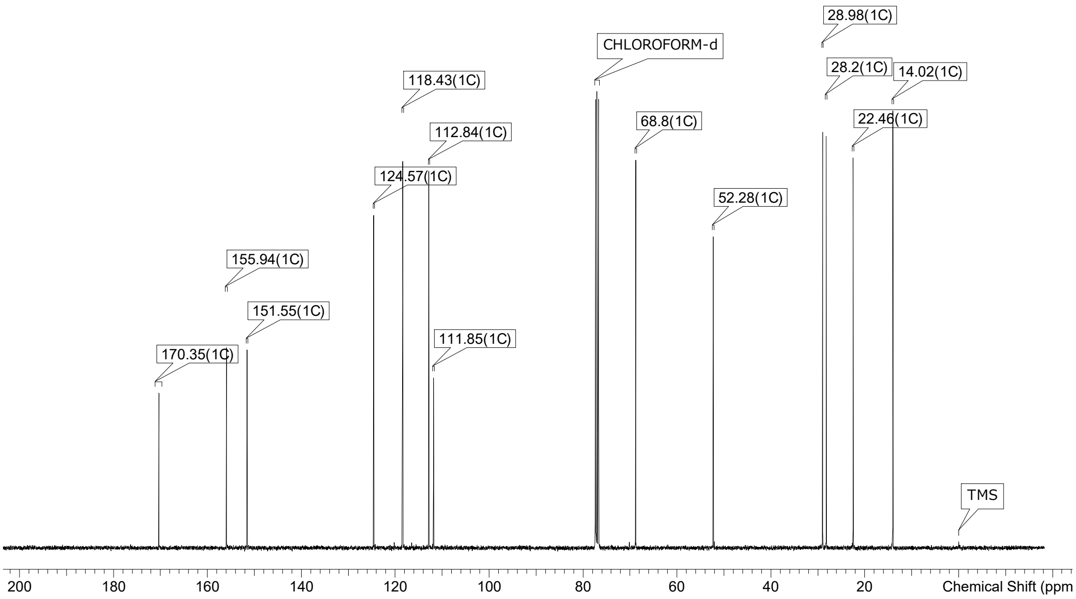

Methyl 5-(hexyloxy)-2-hydroxybenzoate (6)

^{1}H NMR (400 MHz, CHLOROFORM-*d*) δ ppm 0.87 - 0.95 (m, 3 H) 1.34 (br d, *J*=7.25 Hz, 4 H) 1.41 - 1.51 (m, 2 H) 1.69 - 1.83 (m, 2 H) 3.91 (t, *J*=6.57 Hz, 2 H) 3.95 (s, 3 H) 6.90 (d, *J*=9.01 Hz, 1 H) 7.08 (dd, *J*=9.07, 3.06 Hz, 1 H) 7.29 (d, *J*=3.00 Hz, 1 H) 10.33 (s, 1 H)

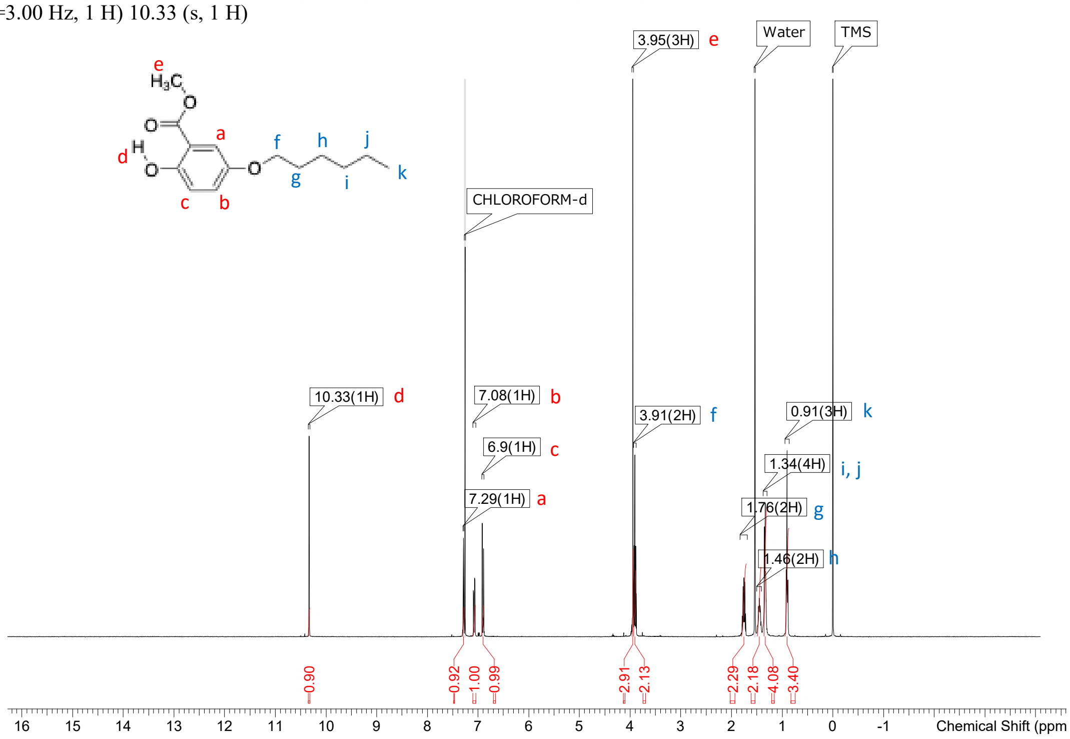

^{13}C NMR (100 MHz, CHLOROFORM-*d*) δ ppm 14.0 (s, 1 C) 22.6 (s, 1 C) 25.7 (s, 1 C) 29.3 (s, 1 C) 31.6 (s, 1 C) 52.3 (s, 1 C) 68.8 (s, 1 C) 111.8 (s, 1 C) 112.8 (s, 1 C) 118.4 (s, 1 C) 124.6 (s, 1 C) 151.6 (s, 1 C) 155.9 (s, 1 C) 170.4 (s, 1 C)

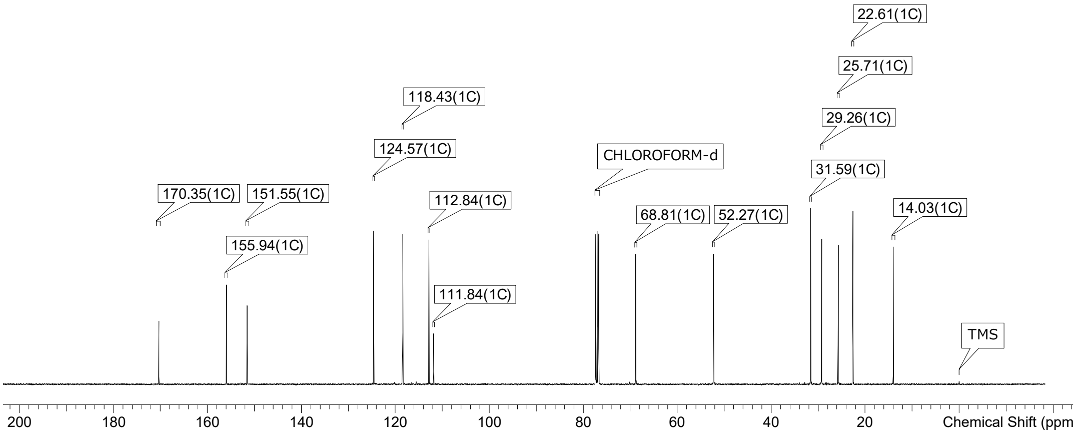

Methyl 5-(heptyloxy)-2-hydroxybenzoate (7)

^{1}H NMR (400 MHz, CHLOROFORM-*d*) δ ppm 0.85 - 0.95 (m, 3 H) 1.27 - 1.39 (m, 6 H) 1.40 - 1.49 (m, 2 H) 1.69 - 1.82 (m, 2 H) 3.91 (t, *J*=6.57 Hz, 2 H) 3.95 (s, 3 H) 6.91 (d, *J*=9.13 Hz, 1 H) 7.08 (dd, *J*=9.01, 3.13 Hz, 1 H) 7.29 (d, *J*=3.00 Hz, 1 H) 10.34 (s, 1 H)

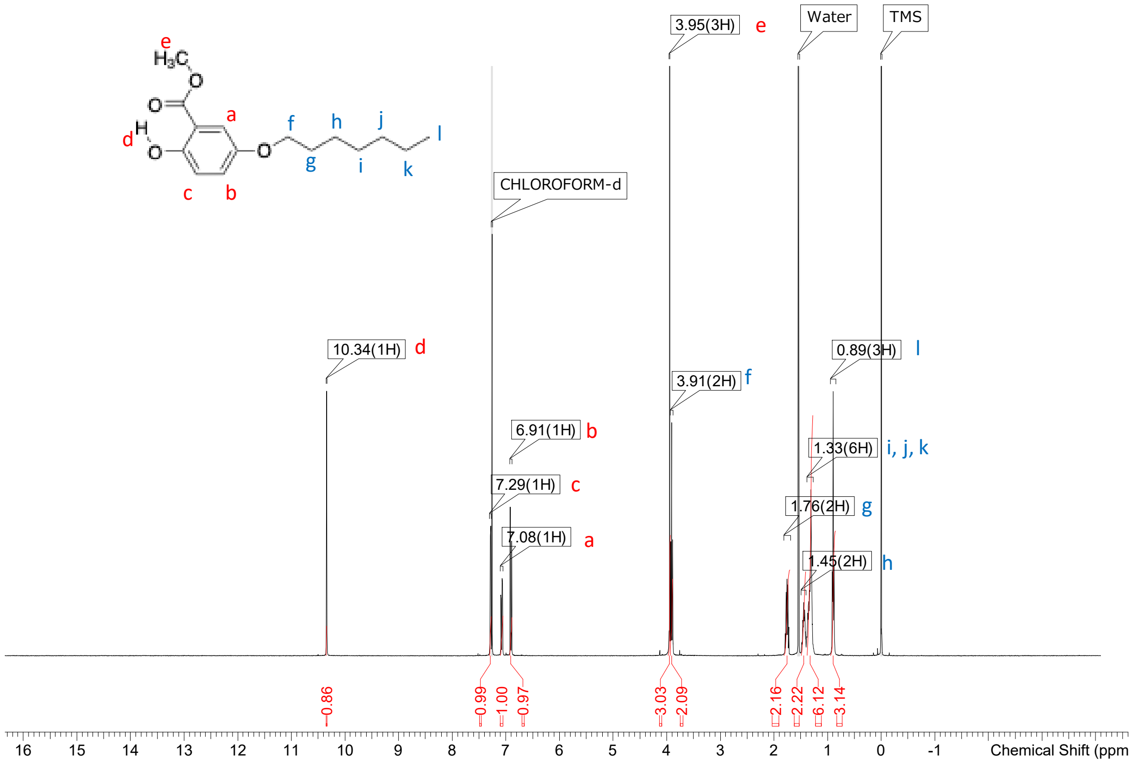

^{13}C NMR (100 MHz, CHLOROFORM-*d*) δ ppm 14.1 (s, 1 C) 22.6 (s, 1 C) 26.0 (s, 1 C) 29.1 (s, 1 C) 29.3 (s, 1 C) 31.8 (s, 1 C) 52.3 (s, 1 C) 68.8 (s, 1 C) 111.6 (s, 1 C) 112.9 (s, 1 C) 118.5 (s, 1 C) 124.6 (s, 1 C) 151.6 (s, 1 C) 155.9 (s, 1 C) 170.4 (s, 1 C)

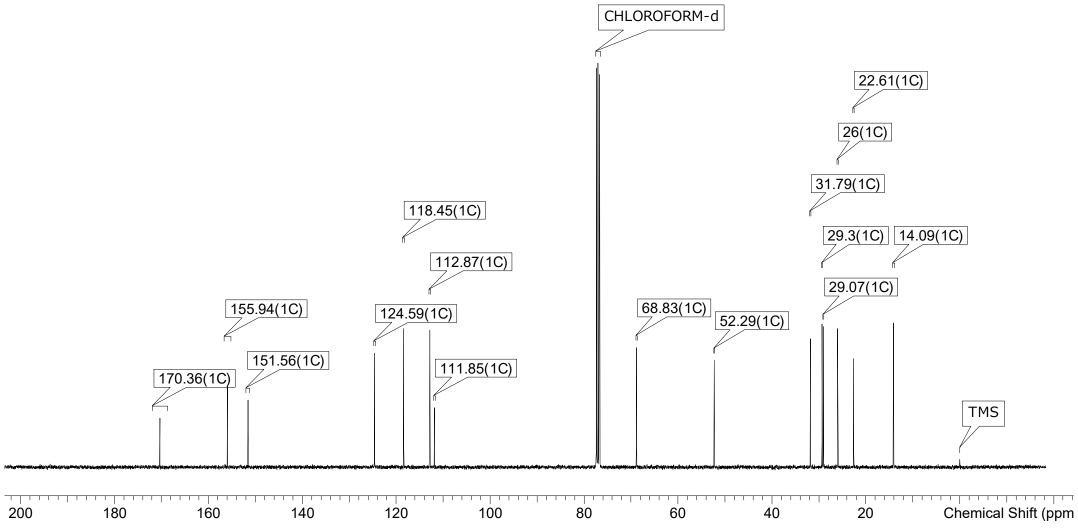

Methyl 2-hydroxy-5-(octyloxy)benzoate (8)

^{1}H NMR (400 MHz, CHLOROFORM-*d*) δ ppm 0.84 - 0.93 (m, 3 H) 1.25 - 1.37 (m, 8 H) 1.39 - 1.50 (m, 2 H) 1.68 - 1.83 (m, 2 H) 3.91 (t, *J*=6.57 Hz, 2 H) 3.95 (s, 3 H) 6.90 (d, *J*=9.01 Hz, 1 H) 7.08 (dd, *J*=9.01, 3.13 Hz, 1 H) 7.29 (d, *J*=3.13 Hz, 1 H) 10.34 (s, 1 H)

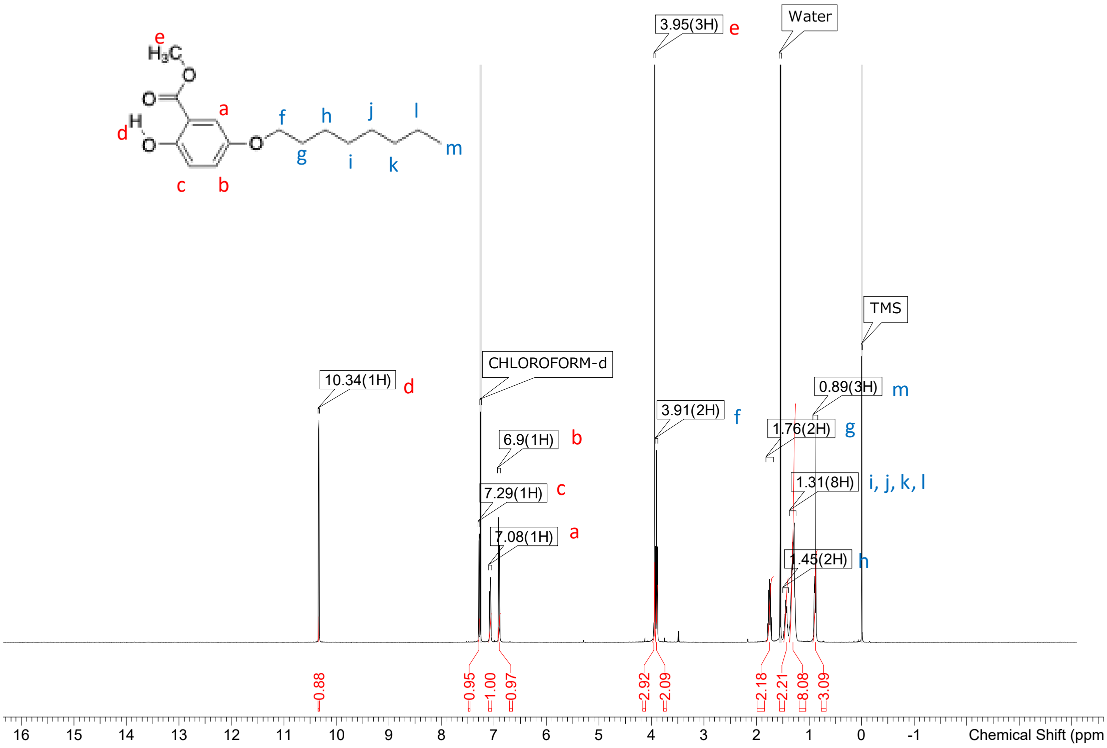

^{13}C NMR (100 MHz, CHLOROFORM-*d*) δ ppm 14.1 (s, 1 C) 22.7 (s, 1 C) 26.0 (s, 1 C) 29.3 (s, 1 C) 29.3 (s, 1 C) 29.4 (s, 1 C) 31.8 (s, 1 C) 52.3 (s, 1 C) 68.8 (s, 1 C) 111.8 (s, 1 C) 112.8 (s, 1 C) 118.4 (s, 1 C) 124.6 (s, 1 C) 151.6 (s, 1 C) 155.9 (s, 1 C) 170.4 (s, 1 C)

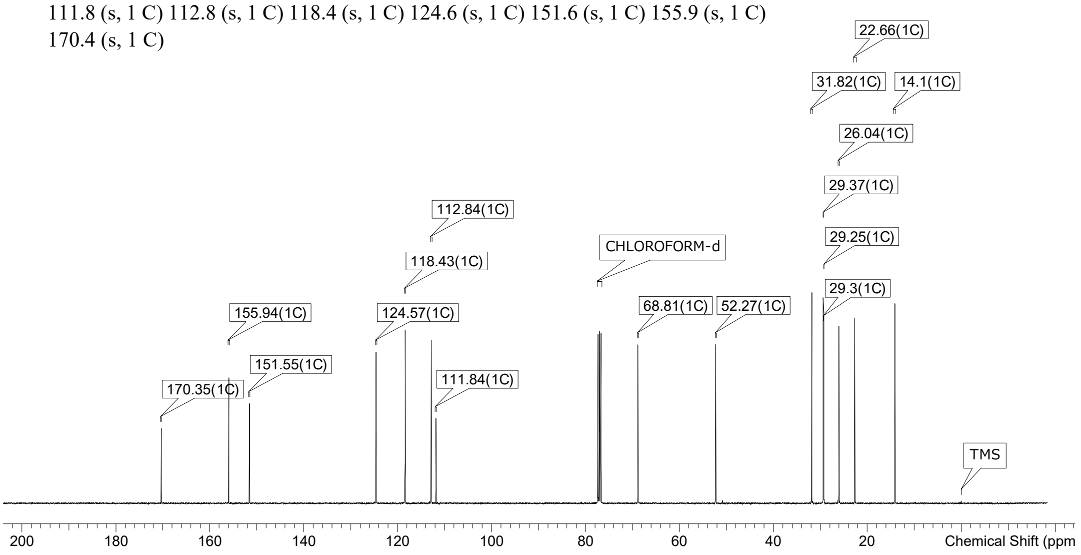

Methyl 2-hydroxy-5-(nonyloxy)benzoate (9)

^{1}H NMR (400 MHz, CHLOROFORM-*d*) δ ppm 0.84 - 0.92 (m, 3 H) 1.25 - 1.39 (m, 10 H) 1.45 (br s, 2 H) 1.69 - 1.82 (m, 2 H) 3.91 (t, *J*=6.57 Hz, 2 H) 3.95 (s, 3 H) 6.90 (d, *J*=9.01 Hz, 1 H) 7.08 (dd, *J*=9.01, 3.13 Hz, 1 H) 7.29 (d, *J*=3.13 Hz, 1 H) 10.34 (s, 1 H)

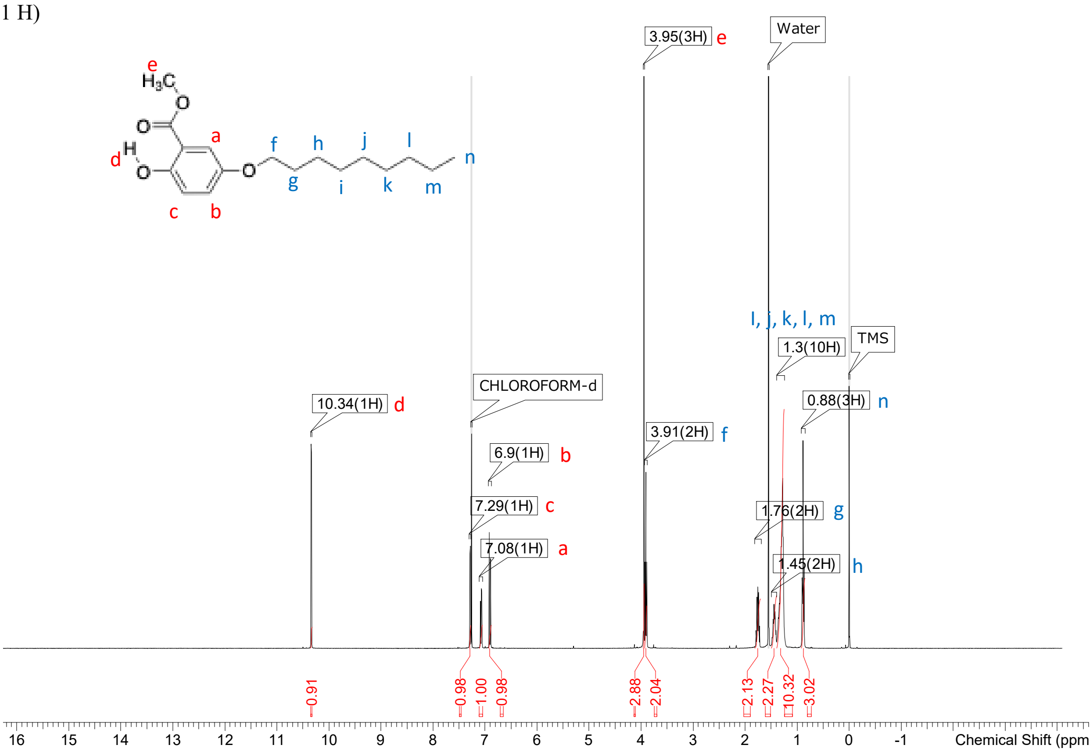

^{13}C NMR (100 MHz, CHLOROFORM-*d*) δ ppm 14.1 (s, 1 C) 22.7 (s, 1 C) 26.0 (s, 1 C) 29.3 (s, 1 C) 29.3 (s, 1 C) 29.4 (s, 1 C) 29.6 (s, 1 C) 31.9 (s, 1 C) 52.3 (s, 1 C) 68.8 (s, 1 C) 111.9 (s, 1 C) 112.9 (s, 1 C) 118.4 (s, 1 C) 124.6 (s, 1 C) 151.6 (s, 1 C) 155.9 (s, 1 C) 170.4 (s, 1 C)

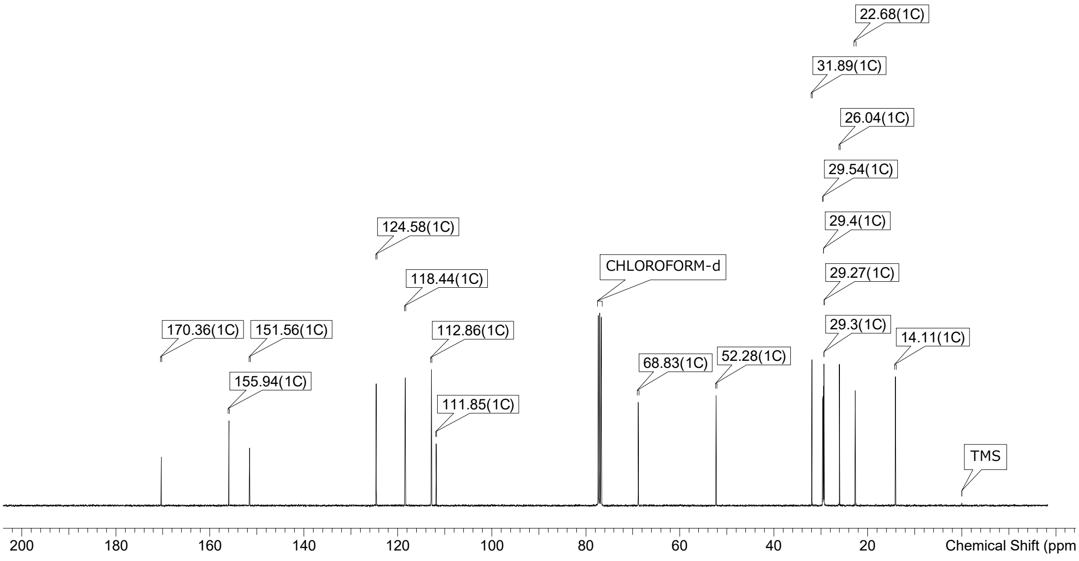

Methyl 5-(decyloxy)-2-hydroxybenzoate (10)

^{1}H NMR (400 MHz, CHLOROFORM-*d*) δ ppm 0.83 - 0.92 (m, 3 H) 1.25 - 1.38 (m, 12 H) 1.39 - 1.50 (m, 3 H) 1.69 - 1.82 (m, 2 H) 3.91 (t, *J*=6.57 Hz, 2 H) 3.95 (s, 3 H) 6.91 (d, *J*=9.01 Hz, 1 H) 7.08 (dd, *J*=9.01, 3.00 Hz, 1 H) 7.29 (d, *J*=3.13 Hz, 1 H) 10.34 (s, 1 H)

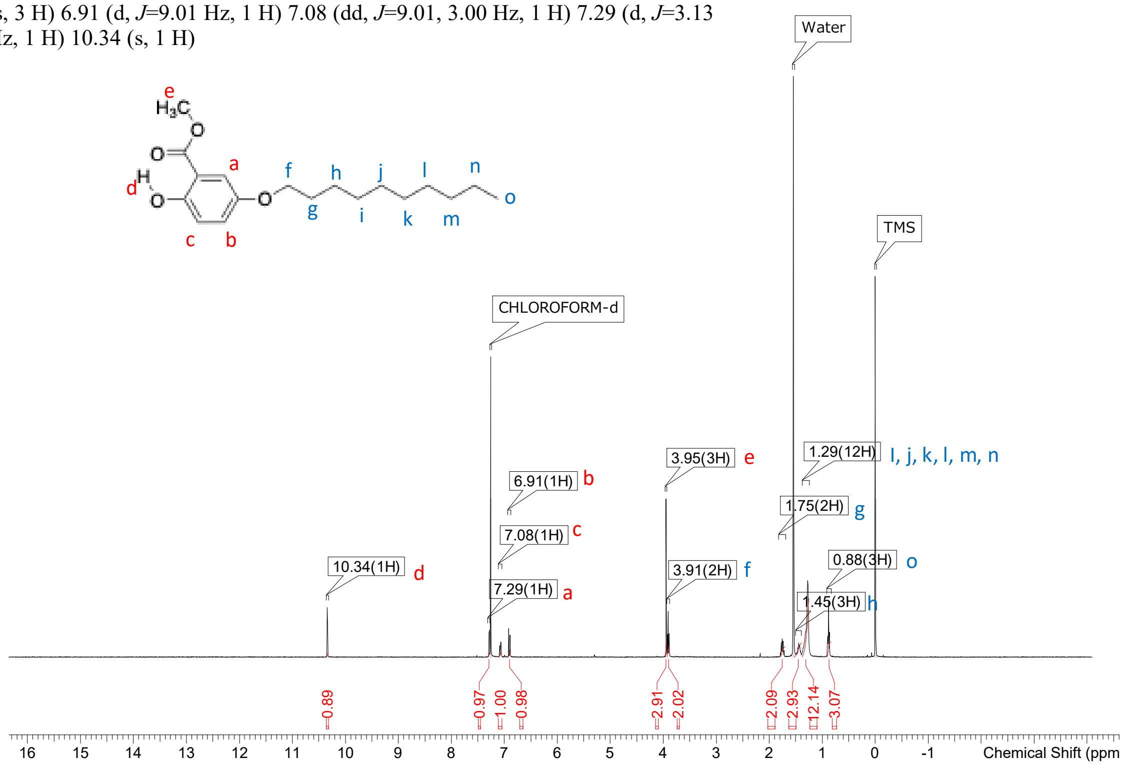

^{13}C NMR (100 MHz, CHLOROFORM-*d*) δ ppm 14.1 (s, 1 C) 22.7 (s, 1 C) 26.0 (s, 1 C) 29.3 (s, 1 C) 29.3 (s, 1 C) 29.4 (s, 1 C) 29.6 (s, 1 C) 29.6 (s, 1 C) 31.9 (s, 1 C) 52.3 (s, 1 C) 68.8 (s, 1 C) 111.9 (s, 1 C) 112.9 (s, 1 C) 118.4 (s, 1 C) 124.6 (s, 1 C) 151.6 (s, 1 C) 155.9 (s, 1 C) 170.4 (s, 1 C)

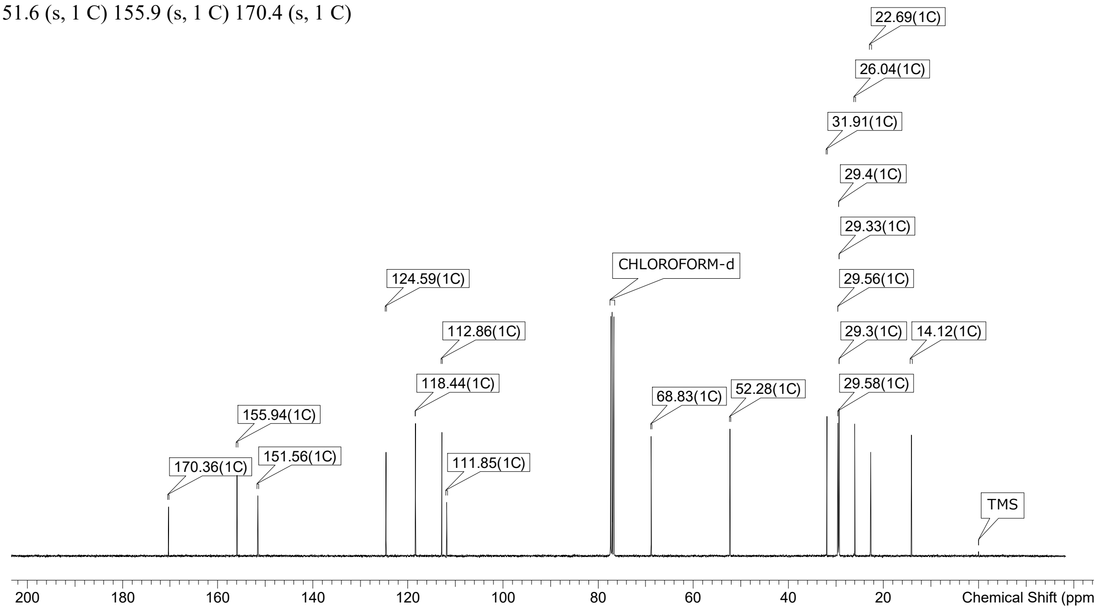

Supplementary Figures and Tables

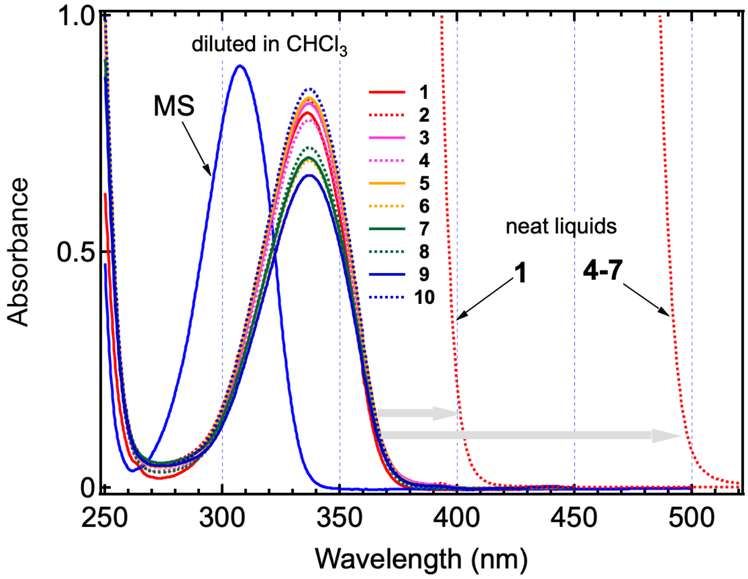

Fig. S1. Absorption spectra of MS and alkoxylated MS derivatives **1**–**10**, diluted with chloroform to an absorbance of approximately 1. The molar concentration was on the order of 10^{-4} M. Red dotted lines on the right indicate the spectral edges of neat colourless liquid **1** and yellow liquids **4**–**7**.

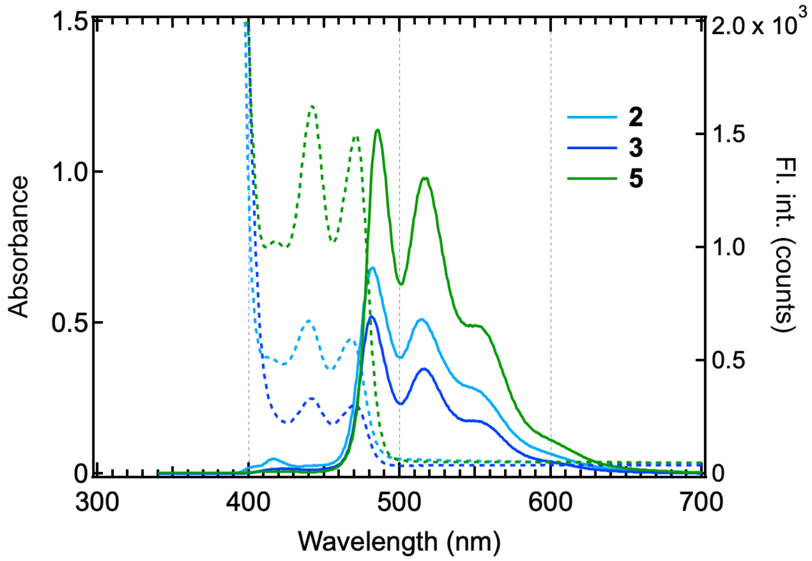

Fig. S2. Absorption and fluorescence spectra of **2**, **3** and **5**, in chloroform solution at 10^{-1} M. λ_{ex} = 330 nm.

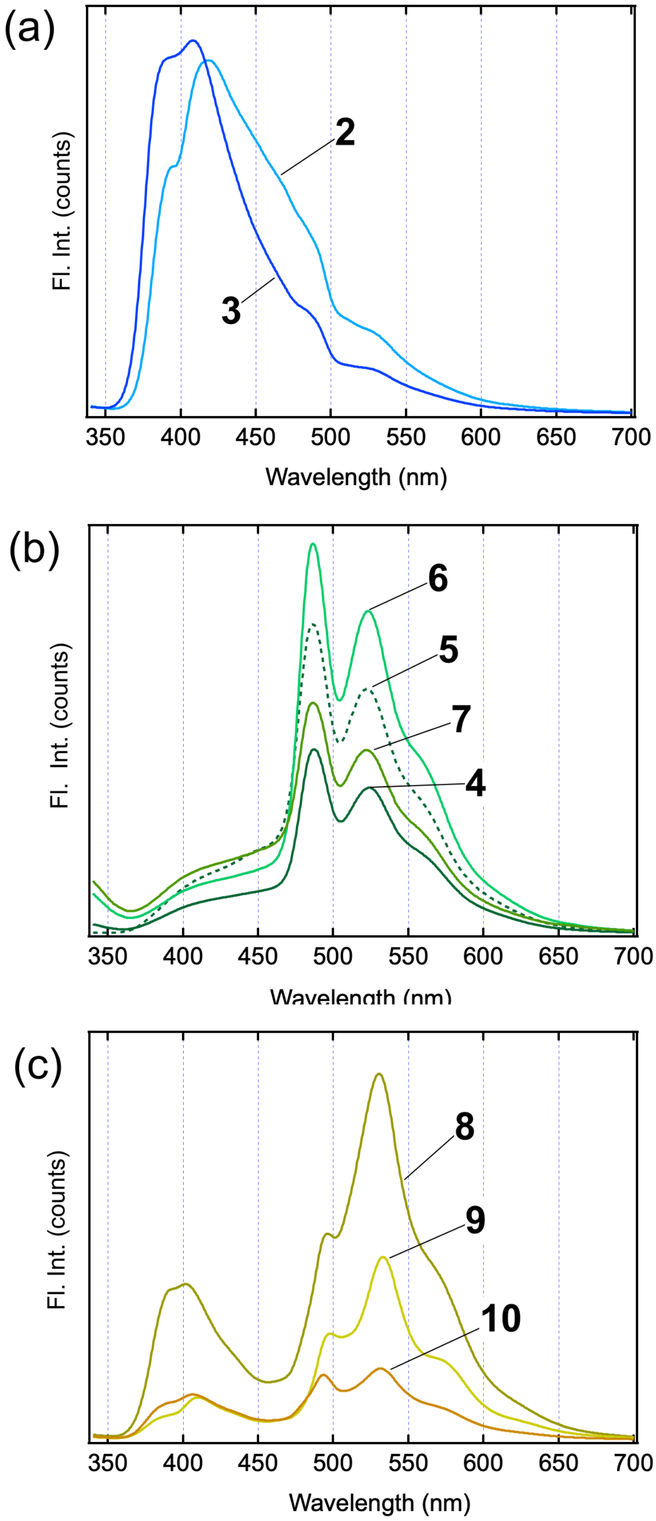

Fig. S3. Fluorescence spectra of **2**–**10** in the neat state. For the liquid samples (**4**–**7**), measurements were performed using a 1 mm path length cell. λ_{ex} = 330 nm.

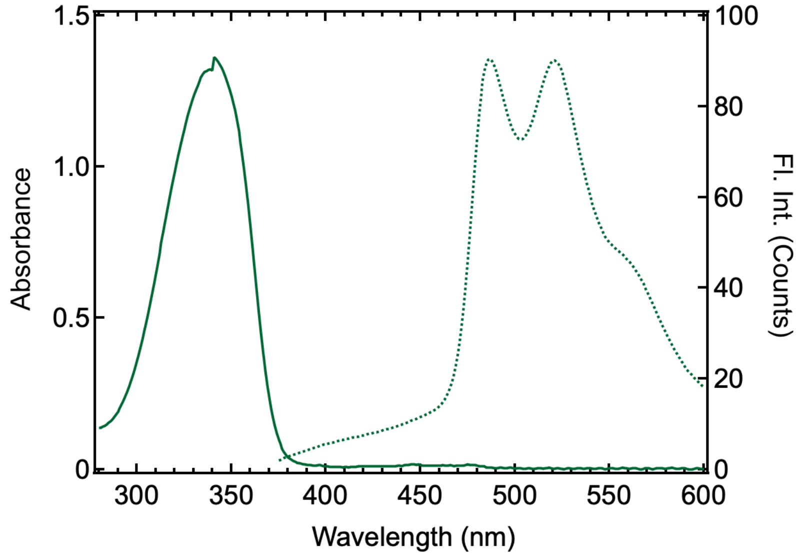

Fig. S4. Absorption (solid line) and fluorescence (dotted line) spectra of neat liquid **5**, measured with the sample sandwiched between quartz plates. λ_{ex} = 365 nm.

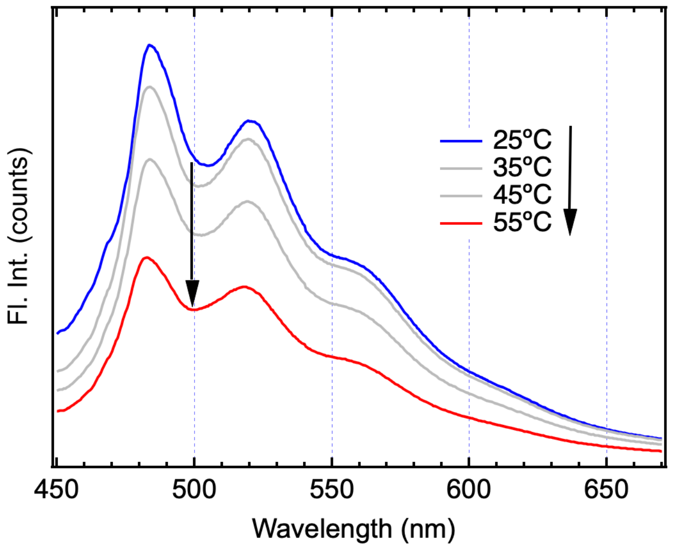

Fig. S5. Fluorescence spectral change of neat liquid **4** upon heating. λ_{ex} = 440 nm.

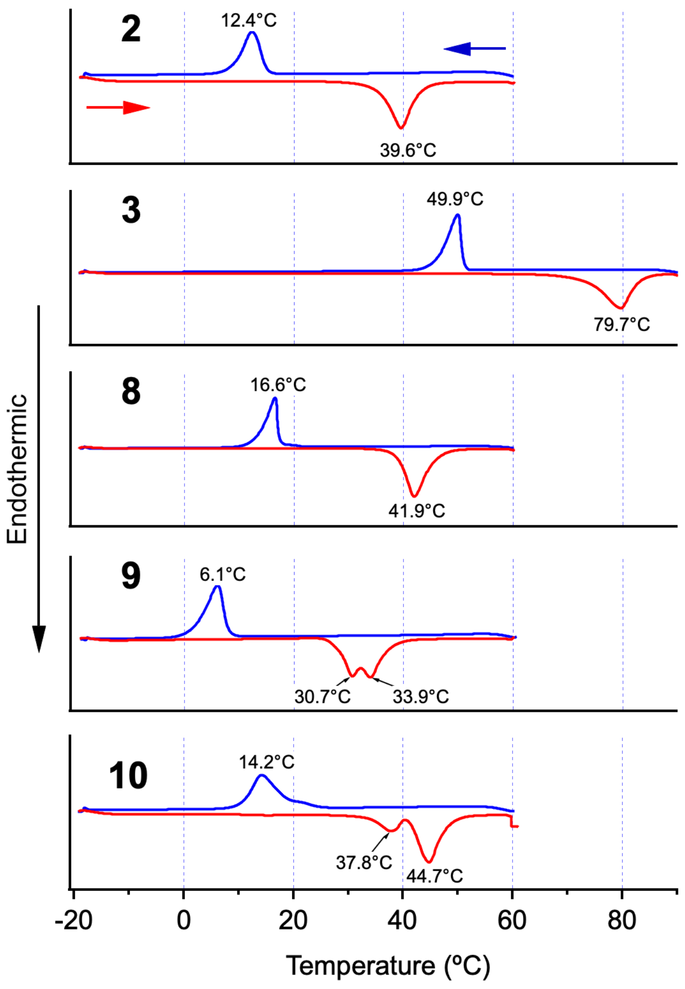

Fig. S6. DSC thermograms of powder samples (**2**, **3**, **8**, **9**, and **10**). Samples were first heated until they melted, and then subjected to cooling and reheating at a rate of 10 °C/min. The cooling and heating processes are represented by blue and red lines, respectively.

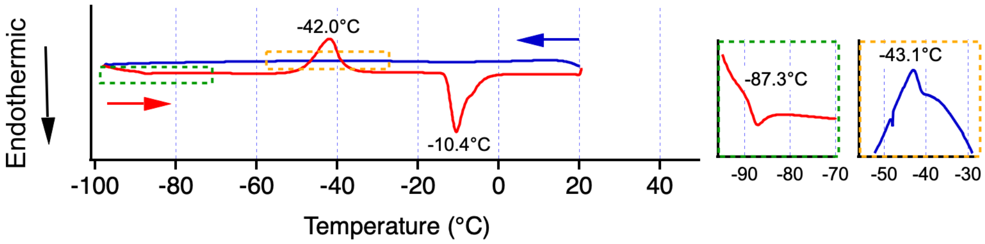

Fig. S7. DSC thermograms of methyl salicylate (MS). The sample was subjected to cooling and heating at a rate of 10 °C/min. The cooling and heating processes are represented by blue and red lines, respectively. The regions with weak peaks (highlighted by green and orange dotted lines) are enlarged and displayed on the right side.

Table S1. Summary of thermal properties determined by DSC measurements.

		Melting				Crystallization			Grass Transition		Cold Crystallization		
**No.**	**State at RT**	**T_{melt} (°C)**		**ΔH_{trans} (kJ/mol)**	**ΔS_{trans} (kJ/mol·K)**	**T_{cryst} (°C)**	**ΔH_{trans} (kJ/mol)**	**ΔS_{trans} (kJ/mol·K)**	**T_{glass} (°C)**	**Specific heats (J/kg·K)**	**T_{cold} (°C)**	**ΔH_{trans} (kJ/mol)**	**ΔS_{trans} (kJ/mol·K)**
**MS**	liquid	-10.4		14.2	54.0				-87.3	110.0	-42.0	-11.5	-49.7
**1**	liquid	17.2		17.2	53.9				-63.8	168.6	-28.7	-10.9	-44.6
**2**	powder	39.6		21.2	67.8	12.4	-19.3	-67.5					
**3**	powder	79.7		30.3	85.8	49.9	-29.9	-92.4					
**4**	liquid	8.6	18.7	12.2	42.0				-67.0	161.9	-0.7	-7.8	-28.6
**5**	liquid	34.7		27.6	89.8				-69.8	223.8	-15.8	-22.7	-88.2
**6**	liquid	21.6		25.7	87.3				-72.3	283.6	-39.5	-14.7	-62.7
**7**	liquid	24.7	35.6	27.7	93.1				-73.7	249.1	-42.3	-14.9	-64.6
**8**	powder	41.9		44.3	140.6	16.6	-40.9	-141.3					
**9**	powder	30.7	33.9	41.2	135.6	6.1	-35.0	-125.4					
**10**	powder	37.8	44.7	41.0	129.0	14.2	-38.9	-135.2					

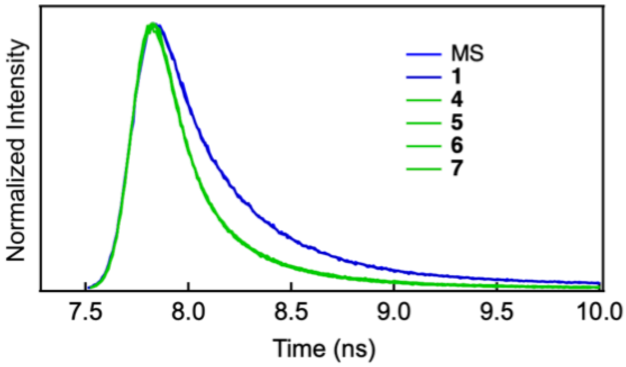

Fig. S8. Positron decay curves of colourless liquids (MS and **1**) and yellow liquids (**4–7).**

Table S2. Lifetime components and their corresponding intensities obtained from the fitting results in **Fig. S2**. Values in parentheses represent the standard errors.

**Compound**	**τ_1**	**I_1**	**τ_2**	**I_2**	**τ_3**	**I_3**
**MS**	0.118 (0.011)	9.639 (0.866)	0.372 (0.004)	69.464 (0.721)	2.479 (0.027)	20.897 (0.271)
**1**	0.153 (0.013)	12.875 (1.620)	0.385 (0.006)	66.246 (1.470)	2.543 (0.025)	20.778 (0.248)
**4**	0.128 (0.002)	44.938 (0.949)	0.368 (0.005)	44.954 (0.846)	2.652 (0.039)	10.108 (0.182)
**5**	0.135 (0.002)	43.344 (0.871)	0.383 (0.006)	43.344 (0.871)	2.707 (0.039)	10.392 (0.186)
**6**	0.133 (0.002)	48.050 (0.967)	0.376 (0.006)	43.074 (0.855)	2.677 (0.047)	8.876 (0.194)
**7**	0.135 (0.002)	49.168 (1.066)	0.369 (0.006)	43.959 (0.938)	2.637 (0.067)	6.873 (0.215)

Table S3. Summary of fluorescence lifetimes for **1–10** in dilute solutions and in the neat state.

		**dilute solutions (3 × 10^{-4} M) in $CHCl_3$**				**Neat samples**			
**comp.**	**state**	**λ_{ex} / nm**	**λ_{em} / nm**	**τ_1 /ns**	**τ_2 /ns**	**λ_{ex} / nm**	**λ_{em} / nm**	**τ_1 /ns**	**τ_2 /ns**
**1**	liquid	375	400	0.5 (94%)	6.7 (6%)	375	450	0.5 (9%)	25.1 (91%)
**2**	powder	375	400	0.5 (93%)	4.2 (7%)	375	400	5.0 (43%)	8.6 (56%)
**3**	powder	375	400	0.5 (95%)	6.1 (5%)	375	400	3.4 (12%)	7.5 (88%)
**4**	liquid	375	400	0.5 (94%)	1.9 (6%)	375	500	3.2 (57%)	15.3 (43%)
**5**	liquid	375	400	0.5 (86%)	3.0 (14%)	375	500	3.4 (67%)	18.9 (33%)
**6**	liquid	375	400	0.5 (94%)	5.0 (6%)	375	500	3.3 (61%)	19.0 (39%)
**7**	liquid	375	400	0.5 (95%)	5.4 (5%)	375	500	3.3 (50%)	23.2 (50%)
**8**	powder	375	400	0.6 (64%)	2.1 (36%)	375	500	2.8 (59%)	7.9 (41%)
**9**	powder	375	400	0.5 (49%)	2.0 (51%)	375	500	2.8 (93%)	7.8 (7%)
**10**	powder	375	400	0.6 (56%)	2.1 (44%)	375	500	2.9 (91%)	8.4 (9%)

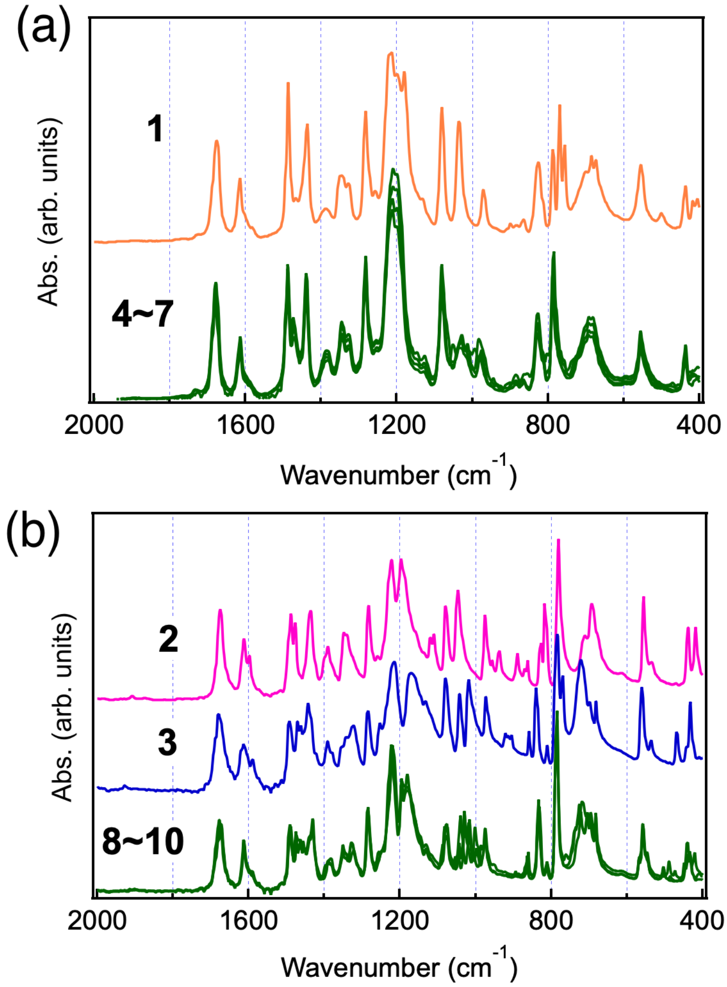

Fig. S9. ATR-IR spectra of MS derivatives: (a) liquid (**1**, **4**–**7**) and (b) powder (**2**, **3**, **8**–**10**) samples.

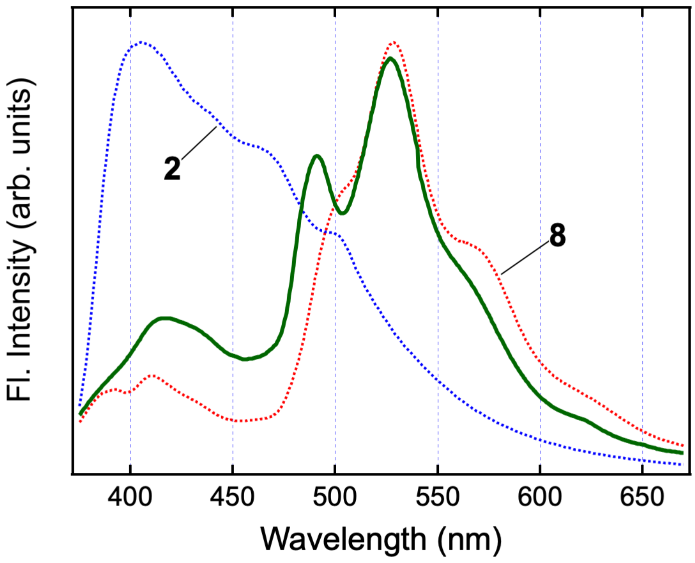

Fig. S10. Fluorescence spectrum of the solid obtained from liquid sample **4** after cold crystallization (green solid line), together with those of solid samples **2** and **8** for comparison. λ_{ex} = 365 nm.

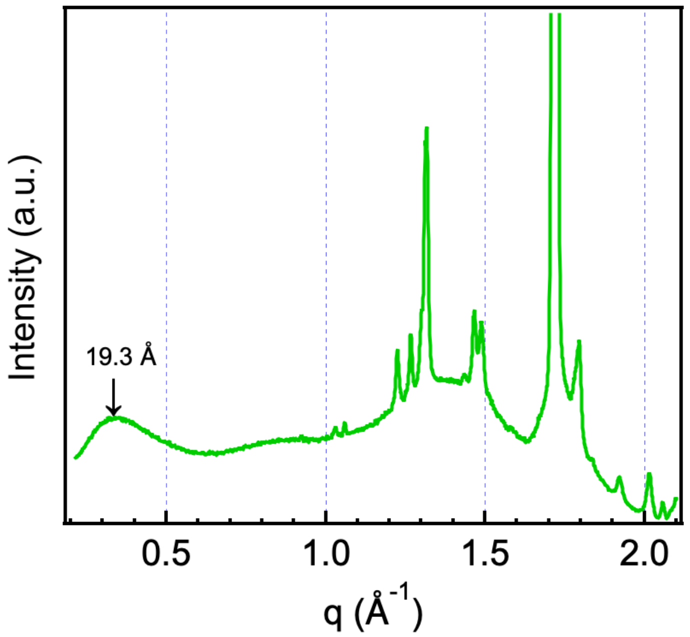

Fig. S11. XRD pattern of **9**, recorded immediately after thermal dissolution.

References

1. Yan, Y.; Qin, B.; Ren, C.; Chen, X.; Yip, Y. K.; Ye, R.; Zhang, D.; Su, H.; Zeng, H., Synthesis, Structural Investigations, Hydrogen−Deuterium Exchange Studies, and Molecular Modeling of Conformationally Stablilized Aromatic Oligoamides, *J. Am. Chem. Soc.*, **2010**, 132, 5869–5879.
